# The Use of a Decision Support System (MyFood) to Assess Dietary Intake Among Free-Living Older Adults in Norway: Evaluation Study

**DOI:** 10.2196/45079

**Published:** 2023-08-03

**Authors:** Frida Severinsen, Lene Frost Andersen, Mari Mohn Paulsen

**Affiliations:** 1 Department of Nutrition Institute of Basic Medical Sciences University of Oslo Oslo Norway

**Keywords:** dietary assessment, malnutrition, eHealth, validation study, older adults, mobile phone

## Abstract

**Background:**

The proportion of older adults in the world is constantly increasing, and malnutrition is a common challenge among the older adults aged ≥65 years. This poses a need for better tools to prevent, assess, and treat malnutrition among older adults. MyFood is a decision support system developed with the intention to prevent and treat malnutrition.

**Objective:**

This study aimed to evaluate the ability of the MyFood app to estimate the intake of energy, protein, fluids, and food and beverage items among free-living older adults aged ≥65 years, primarily at an individual level and secondarily at a group level. In addition, the aim was to measure the experiences of free-living older adults using the app.

**Methods:**

Participants were instructed to record their dietary intake in the MyFood app for 4 consecutive days. In addition, each participant completed two 24-hour recalls, which were used as a reference method to evaluate the dietary assessment function in the MyFood app. Differences in the estimations of energy, protein, fluid, and food groups were analyzed at both the individual and group levels, by comparing the recorded intake in MyFood with the 2 corresponding recalls and by comparing the mean of all 4 recording days with the mean of the 2 recalls, respectively. A short, study-specific questionnaire was used to measure the participants’ experiences with the app.

**Results:**

This study included 35 free-living older adults residing in Norway. Approximately half of the participants had ≥80% agreement between MyFood and the 24-hour recalls for energy intake on both days. For protein and fluids, approximately 60% of the participants had ≥80% agreement on the first day of comparison. Dinner was the meal with the lowest agreement between the methods, at both the individual and group levels. MyFood tended to underestimate the intake of energy, protein, fluid, and food items at both the individual and group levels. The food groups that achieved the greatest agreement between the 2 methods were eggs, yogurt, self-composed dinner, and hot beverages. All participants found the app easy to use, and 74% (26/35) of the participants reported that the app was easy to navigate.

**Conclusions:**

The results showed that the MyFood app tended to underestimate the participants’ dietary intake compared with the 24-hour recalls at both the individual and group levels. The app’s ability to estimate intake within food groups was greater for eggs, yogurt, and self-composed dinner than for spreads, mixed meals, vegetables, and snacks. The app was well accepted among the study participants and may be a useful tool among free-living older adults, given that the users are provided follow-up and support in how to record their dietary intake.

## Introduction

### Background

Globally, the number of people aged ≥65 years is expected to increase considerably in the coming decades [1,2]. Most of the older adults prefer to stay in their own homes, although they experience various illnesses [3], and home care services may contribute to encouraging or enabling individuals to live in their own homes as long as possible [4]. Malnutrition in terms of undernutrition is a condition associated with increased morbidity and mortality risk, reduced quality of life, longer length of hospital stay, and greater economic costs for the health care sector [5-9]. Among home care recipients, malnutrition, or the risk of malnutrition, is common [10-12].

### Guidelines for Nutritional Screening

Guidelines by the European Society for Clinical Nutrition and Metabolism recommend that all older adults should be screened for malnutrition routinely to ensure early identification of risk [13]. According to the European Society for Clinical Nutrition and Metabolism guidelines, individuals found to be malnourished or at risk of malnutrition should receive a comprehensive nutritional assessment and an individualized plan including monitoring and goals for the treatment [13]. With the aim of facilitating dietary assessment, the use of electronic tools in primary health care is emerging, including the use of apps and websites [14].

### The MyFood Decision Support System

MyFood is a digital decision support system consisting of an app for dietary recording and automatic evaluation of the recorded dietary intake as well as a web report for health care professionals including tailored recommendations for nutritional treatment and a nutrition care plan for each patient [15,16]. MyFood was initially developed because of the need for a standardized system to prevent and treat disease-related malnutrition among hospitalized patients in Norway. The dietary assessment functionality in the MyFood system has previously been evaluated in a hospital setting [16], but it has not been validated in other health care settings.

### Objectives

The primary aim of this study was to evaluate the ability of the dietary assessment function in the MyFood app to estimate individual intake of energy, protein, fluid, and food and beverage items among free-living older adults aged ≥65 years at both the individual and group levels using two 24-hour recalls as a reference method. We also aimed to measure the participants’ experiences using the app.

## Methods

### Study Participants and Recruitment

Free-living older adults (aged ≥65 years) were recruited from June 2021 to December 2021 through home care services in a Norwegian municipality, pensioner’s associations, and senior centers. In addition, a web page at the University of Oslo was created with an associated registration form for individuals to express their interest in participation. Finally, participants were recruited by combining convenience sampling and snowball sampling. Eligible participants had to be free-living older adults aged ≥65 years and have a Mini-Mental State Examination–Norwegian Revised (MMSE-NR) score >27. Patients who were terminally ill or psychiatric were excluded from the study.

### The User Interface of the MyFood System

**Figure 1:**
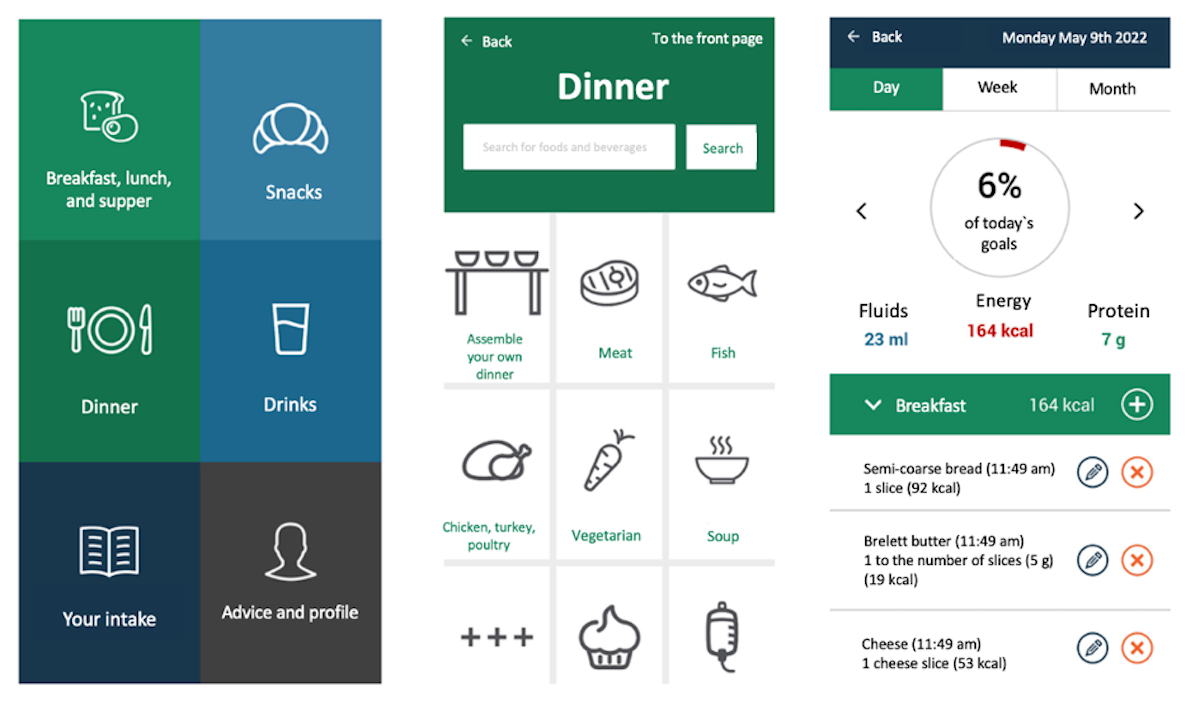
Screenshots of the MyFood app. From left: (1) main menu of the dietary recording function; (2) menu for recording the dinner meal; and (3) evaluation of intake compared with the estimated requirements for energy, protein, and fluid.

MyFood is a decision support system developed by researchers at the University of Oslo and Oslo University Hospital in Norway [16]. The MyFood system includes the following four functions: (1) user registration including anthropometric measures, (2) a dietary recording function, (3) automatic evaluation of recorded nutritional intake, and (4) a report to health care professionals including tailored recommendations for measures to improve nutritional status and a template for a nutrition care plan. The user interface of the MyFood system consists of an app including functions 1 to 3 and a website including function 4. Figure 1 illustrates functions 2 and 3.

### Dietary Recording

Participants were instructed to record their intake in the dietary recording function by selecting 1 of the 5 meal categories (breakfast, lunch, dinner, supper, and snacks). Then, they had to select the correct food or beverage item before recording the amount consumed. Food and beverage items could either be found on the menu or through a free-text search and were illustrated with photographs. When recording dinner intake, the user could choose to select a category of precomposed mixed meals of standardized portions or assemble their own meal using the function *assemble your own dinner* (Figure 1) by selecting all components of the dinner meal manually. During the recording of each meal, the user was presented with prompting questions regarding what proportion of the dish was eaten, whether anything else was eaten with the meal, and whether any beverages or desserts were consumed with the meal.

### Data Collection

The free-living older adults who were recruited as described in the *Methods* section above were contacted by telephone by a project worker, and a suitable time for a visit was agreed upon. At the visit, the participants received written and oral information about the study and signed a consent form. Information on the participants’ age and self-reported height and body weight was retrieved. Participants also completed a Mini Nutritional Assessment (MNA) [17] to assess whether they were at risk of malnutrition and an MMSE-NR [18] to assess whether they had any cognitive impairments that could affect their ability to participate.

All participants were provided guidance on how to download the MyFood app on their personal device, either a tablet or smartphone, except for 2 participants who borrowed tablets available for the project. Then, they were provided with a demonstration of how to use the app. During the demonstration, participants were shown how to navigate the app and how to record their intake of food and beverages.

### Study Design

**Figure 2:**
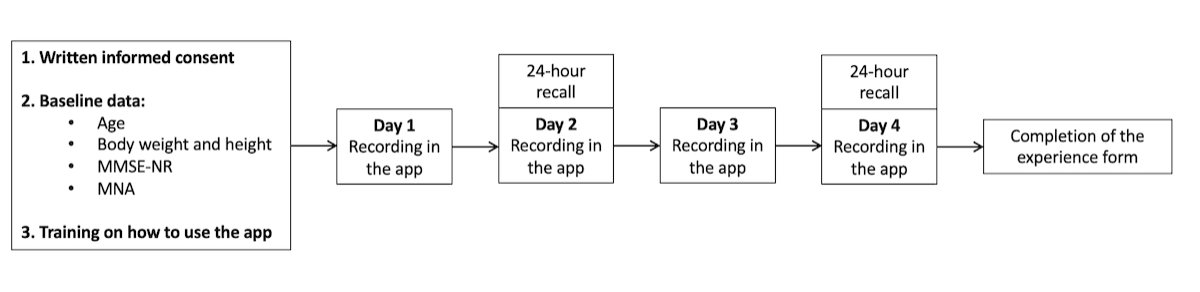
Study design and data collection. MNA: Mini Nutritional Assessment; MMSE-NR: Mini-Mental State Examination–Norwegian Revised.

The participants completed a 4-day recording period, during which they were instructed to record their entire intake of foods and beverages in the MyFood app. As a reference method, all participants completed two 24-hour recalls by phone on 2 days overlapping with the recording period. The overlapping days were on the first and the third day of recording for all participants except for 2, as presented in Figure 2, and are further referred to as “comparison day 1” and “comparison day 2.”

The 24-hour recall procedure used a 3-step sequence within an in-house dietary assessment program (KostBeregningsSystem [KBS]) at the University of Oslo, resembling the US Department of Agriculture’s Automated Multiple-Pass Method [19]. Each recall lasted for approximately 20 to 30 minutes. All participants received a picture booklet to assist in the estimation of portion sizes during the 24-hour recalls, before the registration period. The booklets contained 41 photo series of 4 pictures, in ascending order, of various household measures, food items, and dishes. All food and beverage items recorded in the MyFood app were categorized into 13 food groups: bread and cereals, spreads, eggs, yogurt, cold beverages, hot beverages, self-composed dinner meals, mixed meals, dessert, fruit, vegetables, snacks, and condiments. Mixed meals included all predefined dinner dishes and dinner components recorded without using the function “assemble your own dinner,” and the condiment category included all types of sauces, spices, dressings, etc. The food and beverage items reported in the 24-hour recalls were subsequently allocated to the same categories as the items recorded in the MyFood app for comparison.

### Experience Form

At the end of the 4-day registration period, all participants completed an experience form including 5 claims regarding their perceived usability and applicability of the app, using a 5-point Likert scale ranging from “Strongly disagree” to “Strongly agree.” The content of the experience form was adapted from the System Usability Scale, which is a 10-question scale based on the 5-point Likert scale that provides information about the perceived usability of a digital system [20]. The experience form used in this study has also been used in a previous evaluation study of the MyFood system in a hospital setting [16].

### Sample Size

The sample size estimation was calculated based on the same prerequisites that were used in the previous evaluation study in a hospital setting [21] using a clinically relevant difference between dietary intake recorded in the MyFood app and estimated intake with 24-hour recalls of 50 kcal per day. With a test power of 80%, a significance level of 5%, and a calculated standardized difference of 1, a total of 35 participants were required.

### Data Handling and Statistics

The 24-hour recalls were directly coded into KBS (version 7.4), in which estimations of energy, protein, and fluids were performed using the KBS food composition database AE18. Database AE18 is an extended version of the official Norwegian Food Composition Table (version 2018). Dietary information in the MyFood app for energy (kcal), protein (g), and fluid (mL) was based on the Norwegian Food Composition Table from 2019 and the product information from the manufacturer.

The dietary intake recorded in the MyFood app was compared with the intake reported in the 24-hour recalls. Data were analyzed at both the individual and group levels. Evaluation studies are usually performed at the group level to evaluate tools used in different population groups or settings. As the MyFood system was intended to capture dietary intake at an individual level, the main aim of this study was to evaluate its ability to assess individual intake among the free-living older adults. To be able to use MyFood in groups of older adults, analyses were also included to evaluate the accuracy of the dietary recording function at the group level.

At the individual level, dietary intake data from 2 of the 4 days of dietary recording in the MyFood app were compared with the dietary intake data obtained from the two 24-hour recalls on the corresponding days. The differences in the estimated intake of energy, protein, fluid, and selected food groups between the MyFood app and the 24-hour recalls were presented from the 2 overlapping recording days with both methods. The differences were presented in a series of drop plots for comparison days 1 and 2. In addition, the individual-level data of the differences between the 2 methods were analyzed for the breakfast, lunch, dinner, supper, and snack meals separately, for both comparison days 1 and 2 (Multimedia Appendices 1 and 2). Omitted items were counted as an item mentioned in the recalls but not recorded in the MyFood app.

At the group level, the mean intake from the 4 days of dietary recording in the MyFood app was compared with the mean intake obtained from the two 24-hour recalls. The data were presented with mean and SD. The comparison of the mean intake of energy, protein, and fluid between the 2 methods was analyzed using 2-tailed paired samples *t* tests.

Statistical analyses were performed using SPSS Statistics Software (version 28.0; IBM Corp). The level of statistical significance was set at *P*<.05, and all tests were 2 sided.

### Ethics Approval and Informed Consent

The study was performed in accordance with the Declaration of Helsinki, and the research protocol was reported to The Norwegian Centre for Research Data (reference number: 135175). Informed verbal and written consent were obtained from all the participants.

## Results

### Participants

In total, 35 (13 men and 22 women) free-living older adults aged 65-89 years were included in the analyses. The participants had a median age of 71 years and a mean BMI of 25.4 (SD 4.03) kg/m^2^. Most of the participants (20/35, 57%) had a normal nutritional status according to the MNA screening. The median MMSE-NR score was 29, ranging from 27 to 30, indicating a good cognitive function among the participants. The characteristics of the study participants are presented in Table 1.

**Table 1:** Participant characteristics (N=35).

Characteristics		Participants, n (%)
**Age (years)**		
	65-69	12 (34)
	70-74	11 (31)
	75-79	11 (31)
	80-84	0 (0)
	≥85	1 (2)
**Sex**		
	Male	13 (37)
	Female	22 (63)
**BMI (kg/m^2^)^a^**		
	Underweight: <18.5	0 (0)
	Normal weight: 18.5-24.9	20 (57)
	Overweight: 25-29.9	9 (26)
	Obese: ≥30	6 (17)
**MNA^b^ score**		
	Malnourished: 0-7	0 (0)
	Risk of malnutrition: 8-11	5 (14)
	Normal nutritional status: 12-14	30 (86)
**MMSE^c^ score**		
	0-26	0 (0)
	27-30	35 (100)

^a^Weight (kg)/height (m)^2^.

^b^MNA: Mini Nutritional Assessment.

^c^MMSE-NR: Mini-Mental State Examination–Norwegian Revised.

### Intake of Energy, Protein, and Fluid at the Individual Level

#### Energy Intake

**Figure 3:**
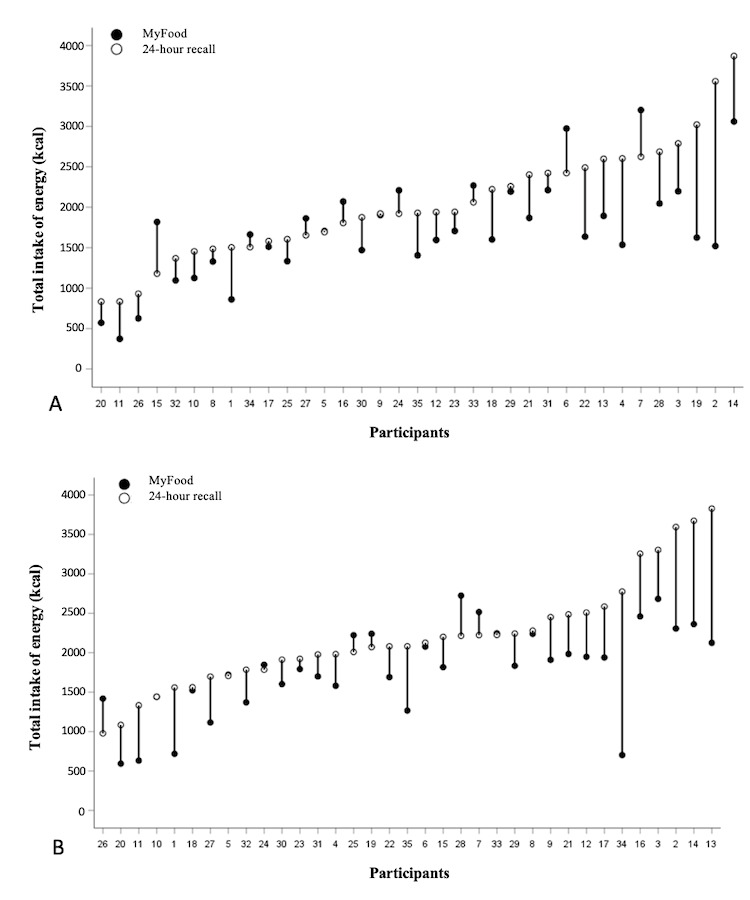
Drop plots illustrating the individual intake of energy on comparison days 1 (section A) and 2 (section B). The y-axis represents the energy intake (kcal). The x-axis represents the participants’ ID numbers. In cases where only a white dot is present, the recorded intake in MyFood was identical to that in the 24-hour recall.

Individual drop plots for the total intake of energy on the 2 comparison days are presented in Figure 3, showing the estimated intake in the MyFood app compared with the 24-hour recalls. The MyFood app tended to underestimate the total intake of energy on both comparison days compared with the 24-hour recalls, and the discrepancies tended to increase with increasing intake of energy.

#### Protein Intake

**Figure 4:**
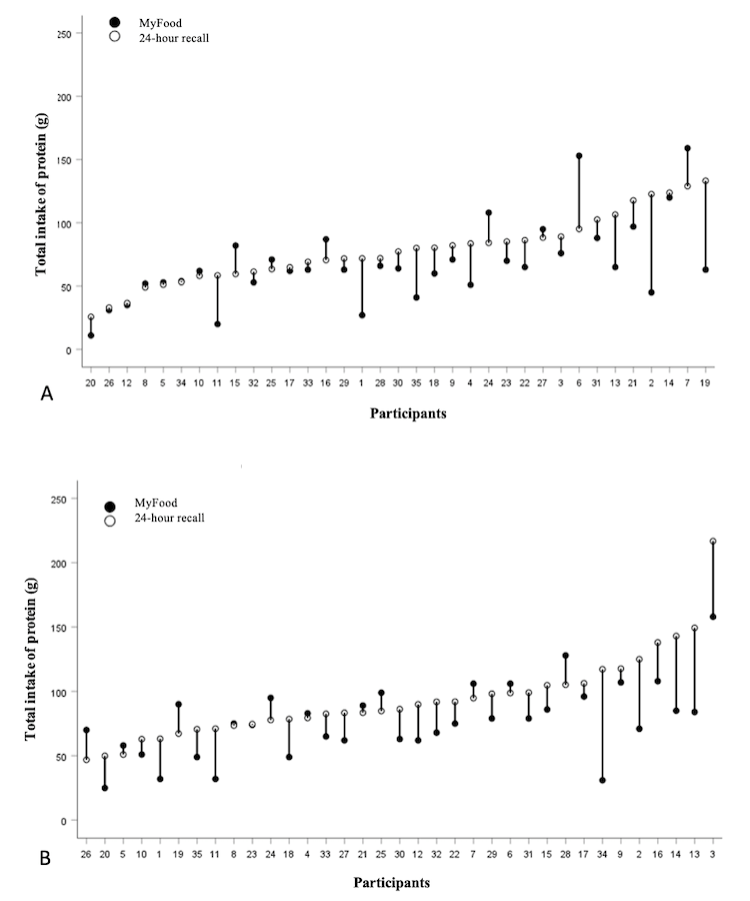
Drop plots illustrating the individual intake of protein on comparison days 1 (section A) and 2 (section B). The y-axis represents the protein intake (g). The x-axis represents the participants’ ID numbers. In cases where only a white dot is present, the recorded intake in MyFood was identical to that in the 24-hour recall.

Individual drop plots for the total intake of protein on the 2 comparison days are presented in Figure 4, showing the estimated intake in the MyFood app and the 24-hour recalls. As for energy, the MyFood app tended to underestimate the intake of protein compared with the 24-hour recalls on both comparison days. The level of discrepancies between the 2 methods seemed to increase with increasing intake of protein.

#### Fluid Intake

**Figure 5:**
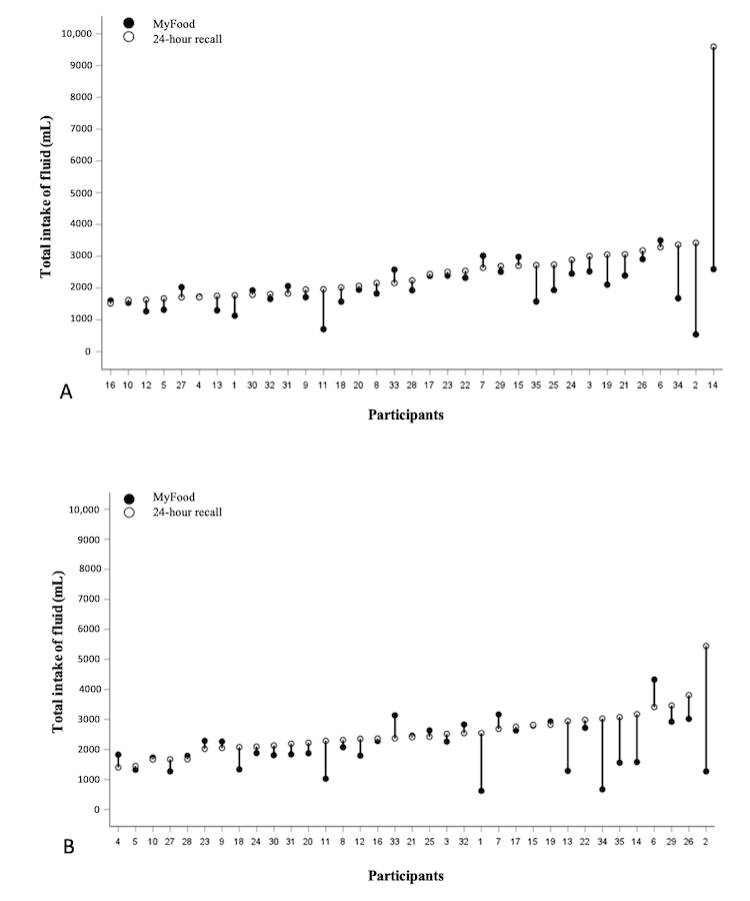
Drop plots illustrating the individual intake of fluids on comparison days 1 (section A) and 2 (section B). The y-axis represents the fluid intake (mL). The x-axis represents the participants’ ID numbers. In cases where only a white dot is present, the recorded intake in MyFood was identical to that in the 24-hour recall.

Individual drop plots for the total intake of fluids on the 2 comparison days are presented in Figure 5, showing the total intake of fluid estimated in MyFood and the 24-hour recalls. For most participants, there was a relatively good agreement between the 2 methods. In cases of discrepancies, the MyFood app mainly underestimated the intake of fluids compared with the 24-hour recalls.

An overview of the proportion of the participants having ≥80% agreement between their recordings in the MyFood app and the intake reported in the 24-hour recalls, in total and for each meal separately, on both comparison days, is presented in Multimedia Appendix 3.

On the first and second comparison day, 49% (17/35) and 51% (18/35) of the participants, respectively, had ≥80% agreement for the total intake of energy. For the total intake of fluids, 63% (22/35) and 60% (21/35) of the participants had ≥80% agreement on comparison days 1 and 2, respectively. For protein, 63% (22/35) of the participants had ≥80% agreement on the first comparison day compared with 46% (16/35) of the participants on the second comparison day.

On the first and second comparison day, 53% (18/34) and 54% (19/34) of the participants had ≥80% agreement for the intake of protein for breakfast, respectively. For lunch, the number of participants having ≥80% agreement on protein intake was somewhat lower, with 48% (14/29) on the first comparison day and 45% (14/30) on the second comparison day.

The dinner was the meal with the lowest proportion of participants having ≥80% agreement between the 2 methods for energy intake, with 41% (14/34) of the participants on the first comparison day and 17% (6/35) of the participants on the second comparison day. The dinner was also the meal that most participants did not record in the MyFood app, with 9% (3/33) of the participants on the first day and 17% (5/30) of the participants on the second day.

The meal with the lowest proportion of participants with ≥80% agreement for fluid intake was snacks, with 27% (9/33) participants on the first comparison day and 25% (8/32) participants on the second comparison day.

### Intake of Energy, Protein, and Fluid at the Group Level

Table 2 presents the mean (SD) intake of energy, protein, and fluid estimated for the 4 days of dietary recording in the MyFood app and the two 24-hour recalls. At the group level, the participants recorded approximately 17% less energy, protein, and fluids in the MyFood app compared with what was reported in the 24-hour recalls, representing approximately 350 kcal, 15 g, and 400 mL, respectively.

**Table 2:** Intake of energy, protein, and fluid presented as mean intake estimated for the 4 days of recording dietary intake with the MyFood app and mean intake estimated from the two 24-hour recalls.

	MyFood (N=35), mean (SD)	24-hour recalls (N=35), mean (SD)	*P* value^a^
Energy (kcal)	1733 (527)	2114 (630)	<.001
Protein (g)	72 (25)	86 (25)	<.001
Fluid (mL)	2017 (719)	2450 (611)	<.001

^a^Differences between the estimated intake recorded in MyFood and the 24-hour recalls were tested using the paired sample *t* test.

### Intake of Food and Beverage Items at the Individual Level

The proportion of participants having ≥80% agreement between their recordings in the MyFood app and the 24-hour recalls within the different food groups on comparison days 1 and 2 varied between the different food groups (Multimedia Appendix 4). Eggs and yogurt were the food groups with the greatest proportion of participants having ≥80% agreement between the 2 methods, with 69% (9/13) and 94% (15/16) of the participants for eggs and 67% (10/15) and 89% (8/9) of the participants for yogurt on days 1 and 2, respectively. The food group with the lowest proportion of participants with ≥80% agreement was condiments. For dinner, the food group “self-composed dinner” achieved better agreement than the food group “mixed meals.”. On the first comparison day, a total of 14 participants used the “assemble your own dinner” function, compared with 9 participants on the second comparison day.

An overview of the omitted food and beverage items is presented in Multimedia Appendix 5. Omitted items were counted as an item mentioned in the recalls but not recorded in the MyFood app. The food groups with the most omissions were cold beverages, condiments, and spreads. Approximately 40 cold beverage items and >20 spreads were reported in the 24-hour recalls but not recorded in the MyFood app.

### Participants’ Experiences Using the MyFood App

All participants reported that the MyFood app was easy to use (Table 3). In total, 74% (26/35) of participants agreed that the app was easy to navigate, and 83% (29/35) of the participants reported that they managed to record the amount of foods and beverages correctly. Moreover, 9% (3/35) of the participants reported that they had to acquire new knowledge to use the app, and 77% (27/35) of the participants reported that they became more aware of their own nutritional requirements.

**Table 3:** Participants’ user experiences (responses from the experience form; N=35).

	Easy to use, n (%)	Easy to navigate, n (%)	Correct amount of foods and beverages recorded, n (%)	New knowledge was acquired to use the app, n (%)	Increased awareness of their own requirements, n (%)
Totally disagree	0 (0)	0 (0)	0 (0)	24 (69)	0 (0)
Slightly disagree	0 (0)	3 (9)	2 (6)	8 (23)	3 (9)
Neutral	0 (0)	6 (17)	4 (11)	0 (0)	5 (14)
Slightly agree	19 (54)	20 (57)	15 (43)	2 (6)	5 (14)
Totally agree	16 (46)	6 (17)	14 (40)	1 (3)	22 (63)

## Discussion

### Principal Findings

This study evaluated the dietary assessment function of the MyFood app among free-living older adults aged ≥65 years residing in Norway. MyFood is intended to be used to assess and monitor the nutritional intake of individuals at risk of malnutrition, and the evaluation of individual intake data was therefore of primary interest. This study found that the MyFood app underestimated the dietary intake of food and beverages at both the individual and group levels. At the individual level, there was a variation in the precision of the recordings between the participants, and the level of underestimation tended to increase with increasing intake. The agreement between the MyFood app and the 24-hour recalls for energy, protein, and fluids was higher for breakfast, lunch, and supper than for dinner and snacks. The food groups with the highest agreement between the 2 methods on both comparison days were eggs, yogurt, and self-composed dinner meals, whereas the food groups with the lowest agreement were condiments, vegetables, mixed meals, and cold beverages. All participants found the app easy to use, and most participants (27/35, 77%) experienced that they became more aware of their own nutritional requirements after 4 days of use.

### The MyFood App’s Ability to Estimate the Intake of Energy, Protein, and Fluid at the Individual Level

To the best of our knowledge, only a few applications for dietary assessment have been developed for use or evaluated among free-living older adults aged ≥65 years [22-25]. Furthermore, most evaluation studies have been performed at the group level, whereas this study mainly intended to evaluate the use of the MyFood app at the individual level, as the purpose of the app is to monitor the nutritional intake of individuals to provide customized nutritional follow-up. Thus, this study provides novel knowledge to the field of using digital tools for nutritional assessment among the free-living older adults.

The MyFood app underestimated the total intake of energy compared with the 24-hour recalls for most participants. An explanation may be that several participants only recorded part of their intake in the app, compared with what they reported in the recalls, possibly because of inaccurate recordings. They may also have forgotten to record their intake in the MyFood app. It has previously been demonstrated that incorrect estimates of portion sizes account for approximately half of the errors in energy intake estimations from dietary records administered using technological devices [26]. During the 24-hour recalls, the participants used a picture booklet to describe their portion sizes, whereas the MyFood app included standardized portion sizes using household measures and illustration photos. In an evaluation study of an app-based food record in Switzerland, Bucher Della Torre et al [27] found that participants tended to choose the app-proposed portions even if their real portions were different. Another possible explanation is the omission of food and beverage items in the MyFood app compared with the 24-hour recalls, such as spreads and cold beverages. This was also seen in the previous evaluation study of the MyFood app among hospitalized patients [16], in which spreads and cold beverages were the food groups with the most omissions. Underreporting of energy intake was also observed in a recent study by Hopstock et al [28], in which a web-based dietary assessment tool was evaluated among Norwegian men and women aged ≥60 years.

The largest discrepancies between the methods in estimated energy intake were found for dinner on both the recording days. This finding is in accordance with observations from the previous evaluation study of the MyFood app [16]. A possible explanation for this may be that some participants forgot to record their dinner in the app or that the predefined meals available in the app did not represent the meals that the participants would eat for dinner, as these meals were adapted to an institutional setting and not tailored for a home setting. We observed that the participants who used the function “assemble your own dinner” (Figure 1) achieved better agreement between the 2 methods in energy intake for dinner than those selecting predefined meals in the app. This was possibly a result of them being forced to manually record all meal items. Thus, they could not lean on prerecorded items, which may explain why the “assemble your own dinner” function achieved greater accuracy than the predefined dinner meals. Although less than half of the participants used this function on each comparison day, with only 14 participants on the first day and 9 on the second day, this knowledge will be used in the future development of the dinner recording function in the MyFood app.

The agreement between the 2 methods for the estimated intake of energy, protein, and fluids was greater for participants with low intakes, with increased deviations observed with higher intakes on both comparison days. This corresponds to previous findings of underestimation of protein and fluids in MyFood in a hospital setting [16]. Other studies have shown that adults tend to underestimate large portion sizes compared with smaller ones [29]. The underestimation of protein in the MyFood app may have been caused by the omission of spreads (Multimedia Appendix 5). As sliced bread with spreads such as cheese and ham is often consumed for breakfast and lunch in Norway, spreads are an important source of protein in the Norwegian diet. Spreads were also found to be one of the food groups most often omitted in a Canadian validation study of an automated web-based 24-hour dietary recall using fully controlled feeding studies as the reference method [29]. Fluid intake was also underestimated in the MyFood app. This may have been because of the high omission rate for beverages, causing the reported intake of beverages in the recalls to be greater than those recorded in the app. Another possible explanation is the overestimation of fluid intake, as shown by the very high reported intake for some of the participants in the 24-hour recalls. For each of the meals separately, the agreement between the 2 methods for fluids was poor, with snacks being the meal category with the lowest agreement. This may have been because of the drinks not being recorded together with the meal with which they were consumed but rather being recorded as part of the snack category.

For energy, protein, and fluids, there was a tendency for better agreement between the methods on comparison day 1 than on comparison day 2. This contradicts previous studies, including the former evaluation study on MyFood [16], which demonstrated a “learning effect,” with an improved agreement on the second recording day compared with the first recording day [30].

### MyFood’s Ability to Estimate the Intake of Energy, Protein, and Fluid at the Group Level

The estimated mean total intake of energy, protein, and fluid was underestimated in the MyFood app. A recent systematic review and meta-analysis by Zhang et al [31] on dietary assessment apps found that all apps underestimated energy intake compared with their reference methods. Zhang et al [31] argued that conducting 24-hour recalls the day after using the app might cause a memory effect and reduce the extent of underreporting in the recalls compared with recording in the app. Moreover, the availability of feedback and advice in the app may positively affect the 24-hour recalls performed afterward [32]. In this study, the two 24-hour recalls were conducted after recording in the MyFood app asking the participants to report on the exact same days. This may have led to improved memory and precision in the recalls compared with the recordings in the app.

### MyFood’s Ability to Estimate Intake in Food Groups

The food groups that showed the best agreement between the methods were eggs, yogurt, and self-composed dinners. This may be because eggs and yogurt are presented in standardized units in the app, such as 1 egg or 1 cup of yogurt, which are similar to the units available in the grocery store. We also observed that the meals in which the participants assembled the dishes themselves, that is, breakfast, lunch, and self-composed dinners, achieved greater agreement than the predefined meals, such as mixed meals. For spreads, the agreement between the methods was quite low, which may be a result of spreads being among the food groups with the most omissions, as described in the *Results* section. However, the low agreement between the 2 methods for the spreads in this study may also be because of participants only recording part of the spreads they put on their bread slices or because they had difficulties estimating the correct amount of spreads eaten.

### User Experiences

All participants reported that the MyFood app was easy to use and 74% (26/35) reported that it was easy to navigate. In addition, most participants (27/35, 77%) reported that they became more aware of their own nutritional requirements after using the MyFood app. This corresponds with the findings of the previous evaluation study of the MyFood app in a hospital setting [16]. Most participants (32/35, 91%) responded that they did not have to acquire a lot of new knowledge to use the app. This contradicts the findings of a study by Hopstock et al [28], in which they found that about one-third of the participating Norwegian men and women aged 60-74 years experienced that they needed to learn a lot of things before using a digital tool for dietary assessment. However, in the study by Hopstock et al [28], the participants did not receive any guidance on using the tool, in contrast with this study.

### Strengths and Limitations

This study evaluated the dietary assessment functionality of the MyFood app among free-living older adults, which is considered an important strength, as most studies investigate the use of apps as dietary assessment tools among younger individuals. In addition to evaluating the dietary assessment functionality of the MyFood app, the participants’ experiences with using the app were investigated. Data on the usability of dietary assessment apps among the free-living older adults are scarce.

A limitation of using 24-hour recalls as the reference method is that the 24-hour recalls are prone to error, such as underestimation of intake, which may have affected the basis of comparison, as both the reference method and the test method inhabit the same measurement error [33,34]. The dietary recording functionality of the MyFood app was evaluated in free-living older adults, most of whom did not receive home care services and were not at risk of malnutrition according to the MNA. Thus, the study sample was probably healthier than what may be expected from the general population of free-living older adults aged ≥65 years. Furthermore, many of the participants were still working, and thus, they had a busier everyday life than what many free-living older adults are expected to have. Therefore, we do not know whether the results are representative of the free-living older adult population in Norway. The results indicate that free-living older adults need follow-up to be able to record accurate portion sizes and to avoid omissions in the MyFood app, and future studies should investigate how health care professionals or next-of-kin may be involved in this task.

### Conclusions

The MyFood app was evaluated for its ability to estimate the intake of energy, protein, fluid, and food and beverage items among free-living older adults aged ≥65 years residing in Norway. The results showed that the MyFood app underestimated the participants’ dietary intake compared with 24-hour recalls as a reference method, both at the individual and group levels. The breakfast and the lunch meals showed better agreement between the methods than the dinner and snack meals. The MyFood app may be a useful tool among free-living older adults; however, the results indicate that the free-living older adults need follow-up and support to accurately report portion sizes and avoid omissions. All participants found the MyFood app easy to use, 74% (26/35) found it easy to navigate, and most participants (27/35, 77%) reported becoming more aware of their nutritional requirements.

## Appendix

Multimedia Appendix 1 Drop plots for the intake of energy, protein, and fluids for each meal on comparison day 1.

Multimedia Appendix 2 Drop plots for the intake of energy, protein, and fluids for each meal on comparison day 2.

Multimedia Appendix 3 An overview of the proportion of participants having ≥80% agreement between MyFood and the 24-hour recalls for the estimated intake of energy, protein, and fluid in total and for each meal on comparison days 1 and 2. n1: number of consumers on the first comparison day. n2: number of consumers on the second comparison day.

Multimedia Appendix 4 An overview of participants having ≥80% agreement between MyFood and the 24-hour recalls in the estimated intake of selected food groups on comparison day 1 and day 2. n1: number of consumers comparison day 1. n2: number of consumers comparison day 2.

Multimedia Appendix 5 Number of omitted food items in each food group for comparison days 1 and 2. The light gray bar represents the number of omissions on the first comparison day, and the dark gray bar represents the number of omissions on the second comparison day. In cases where no bars were present, there were no omissions on that day.

## Abbreviations

KBS: KostBeregningsSystem
MMSE-NR: Mini-Mental State Examination–Norwegian Revised
MNA: Mini Nutritional Assessment

## Data Availability

The data sets used and analyzed during this study are available from the corresponding author upon reasonable request.
