# Supplementary figures and images for: The Use of a Decision Support System (MyFood) to Assess Dietary Intake Among Free-Living Older Adults in Norway: Evaluation Study
Source: JMIR Mhealth Uhealth. 2023 Aug 3;11:e45079. doi: 10.2196/45079 (PMC10436117; doi:10.2196/45079)

Drop-plots for intake of energy, protein, and fluids for each meal on comparison day 1


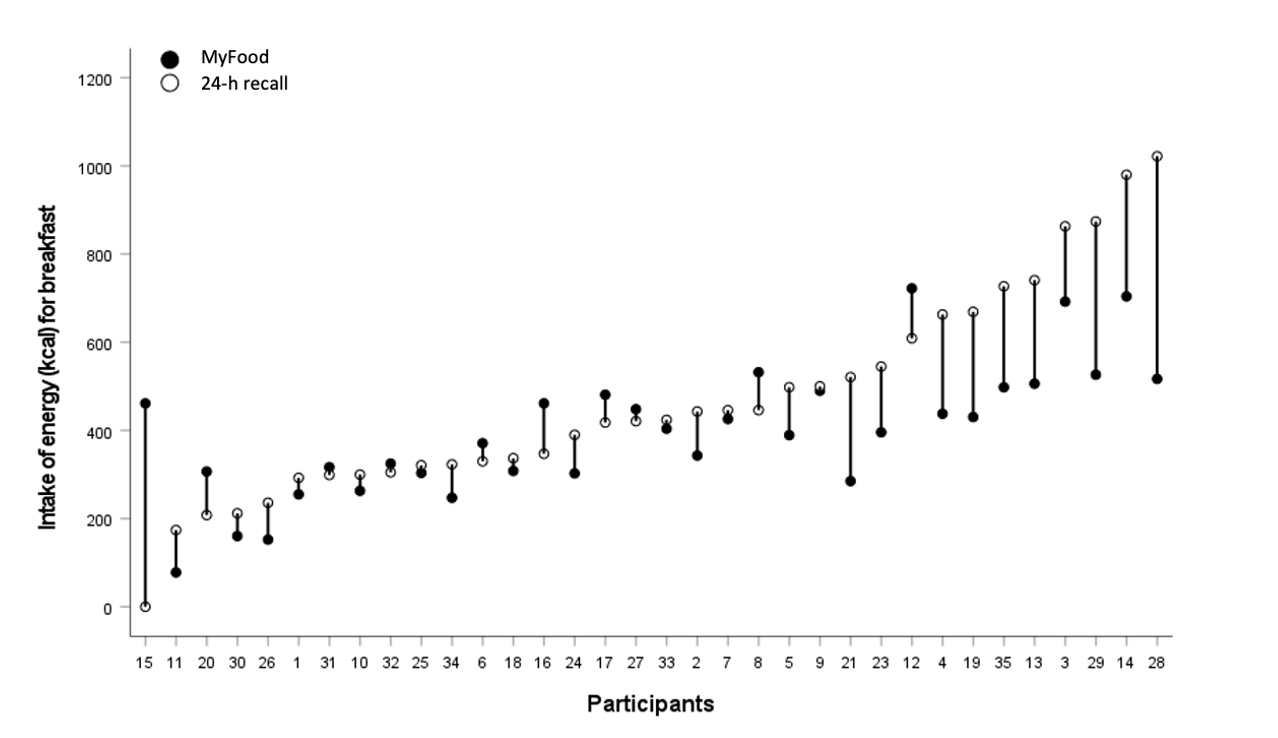


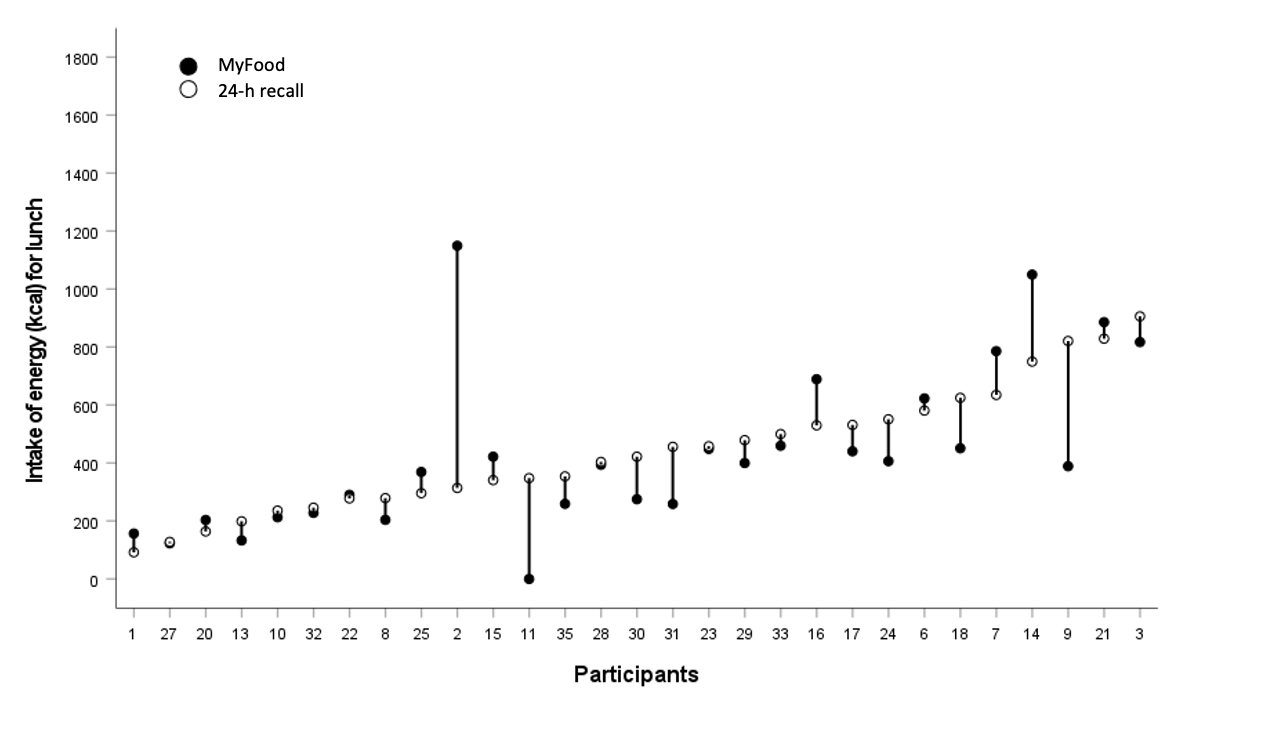


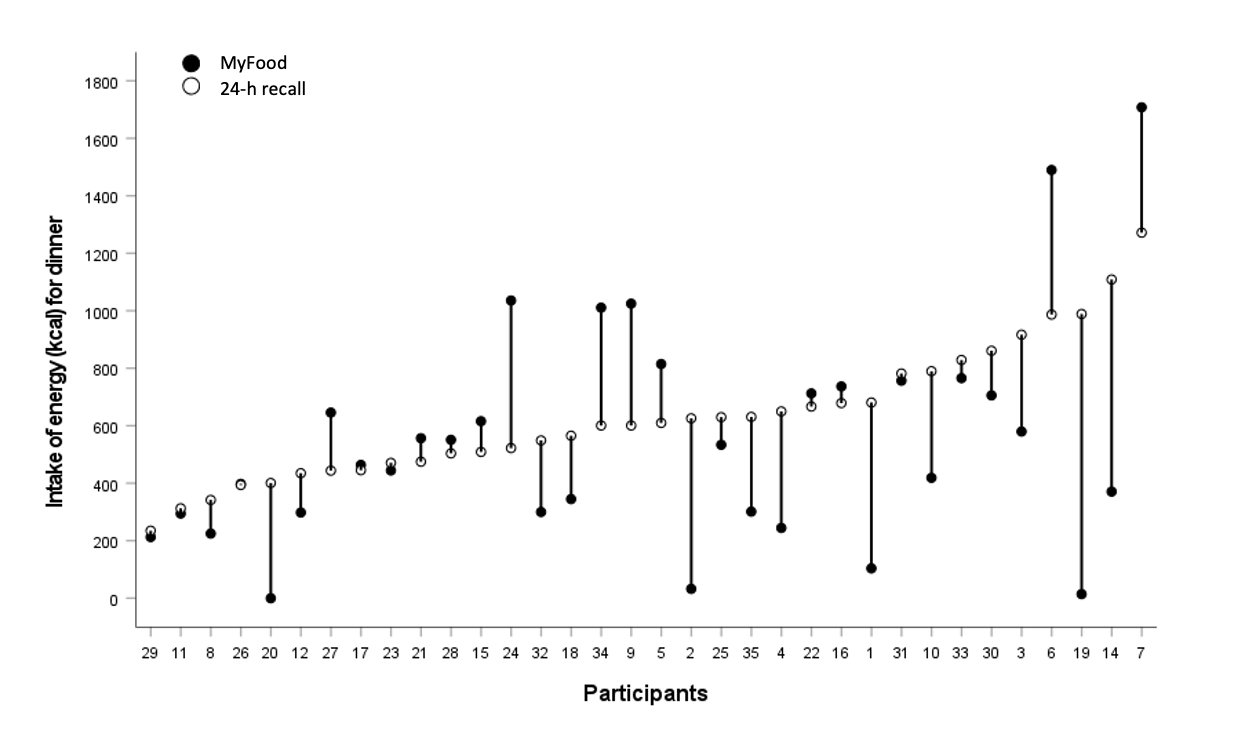


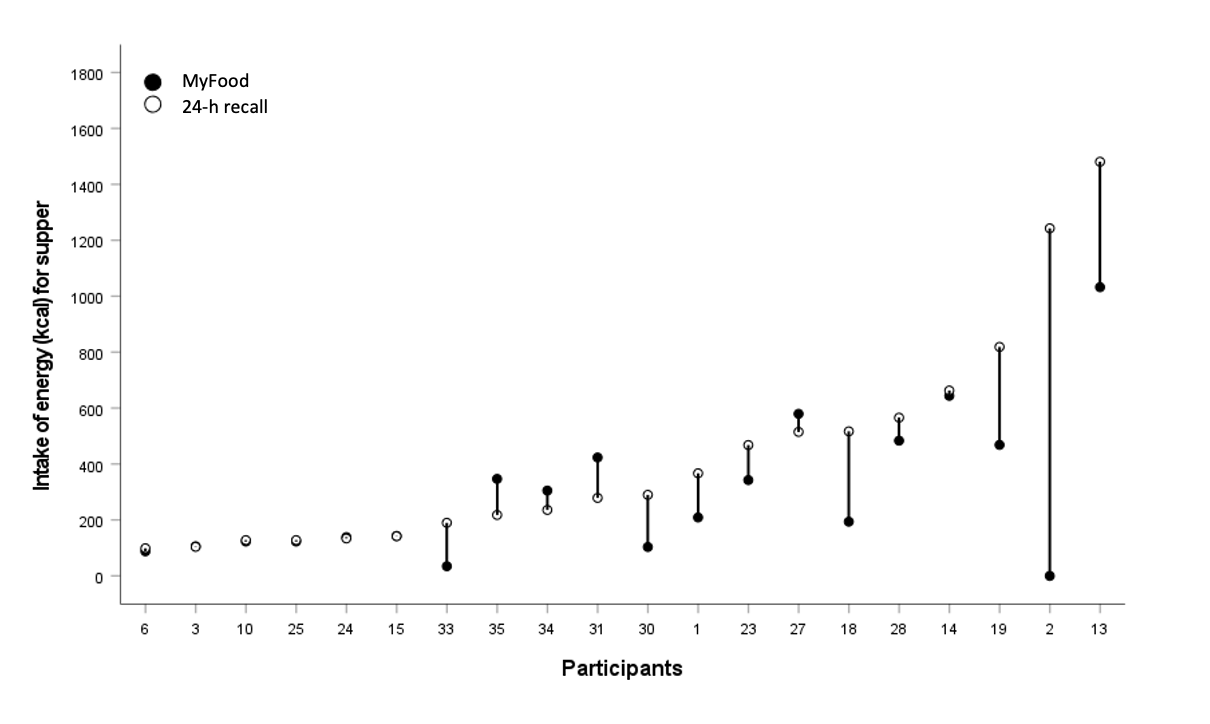


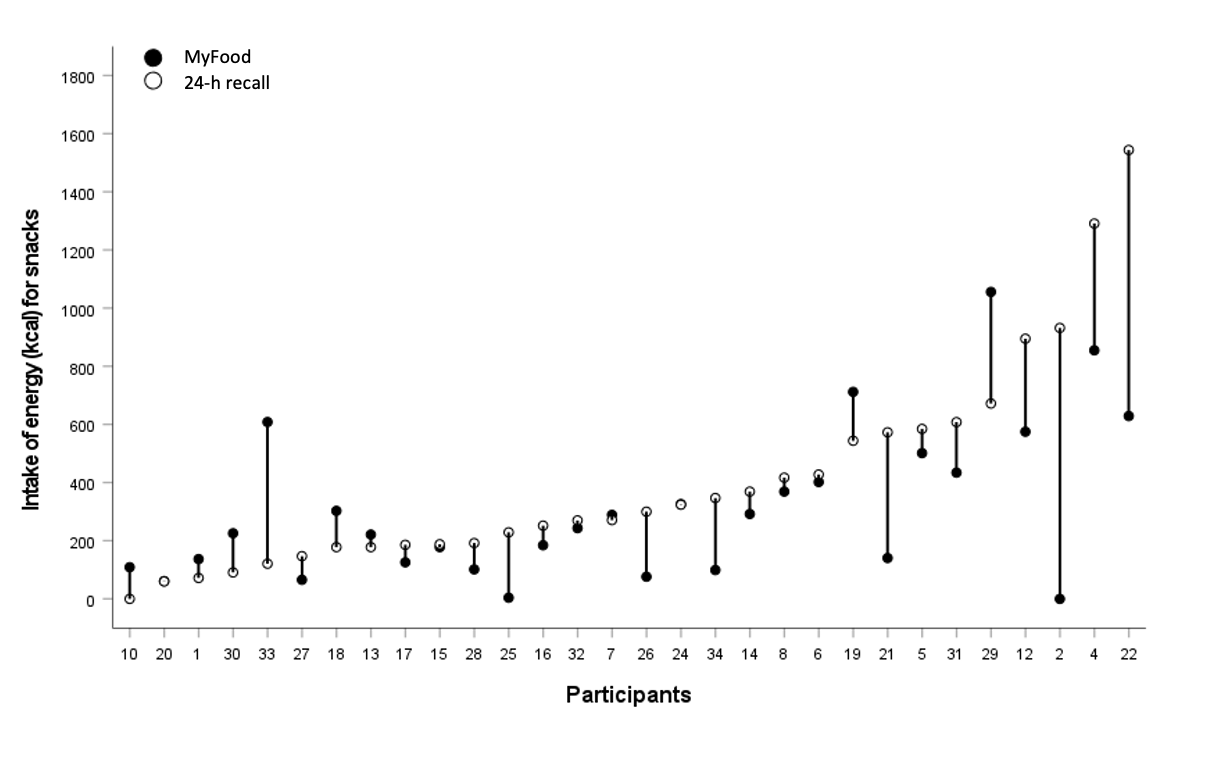


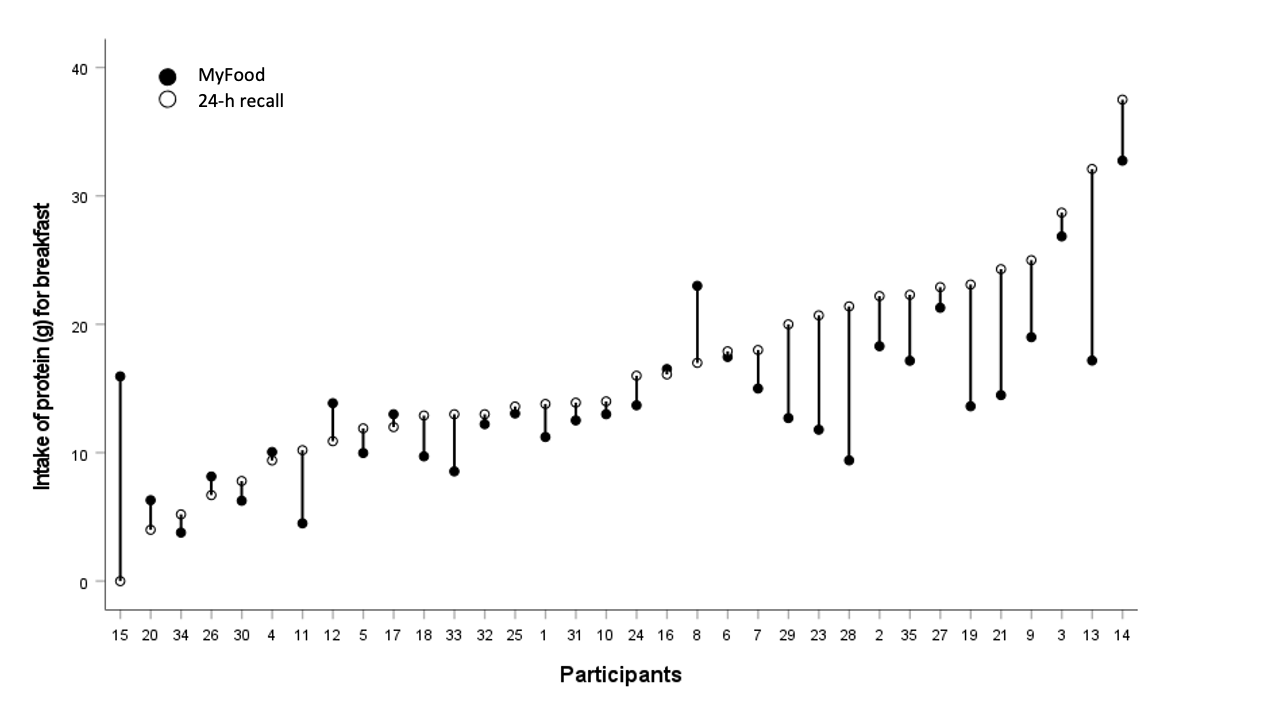


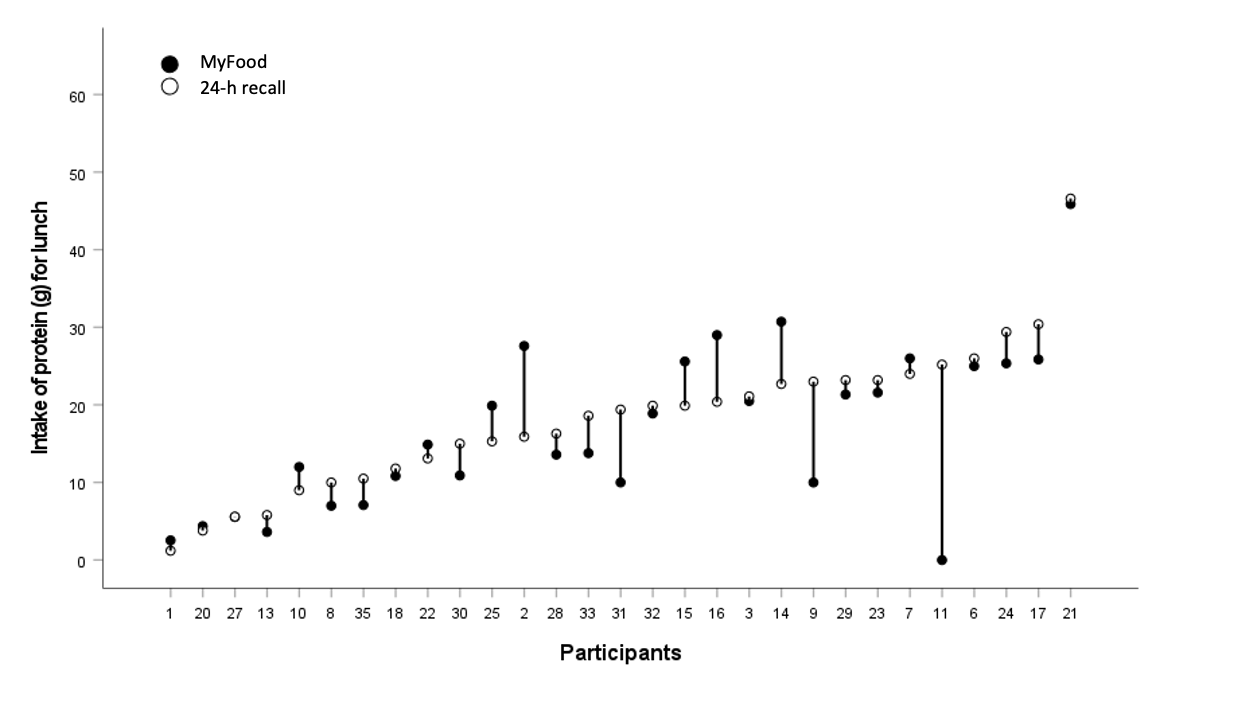


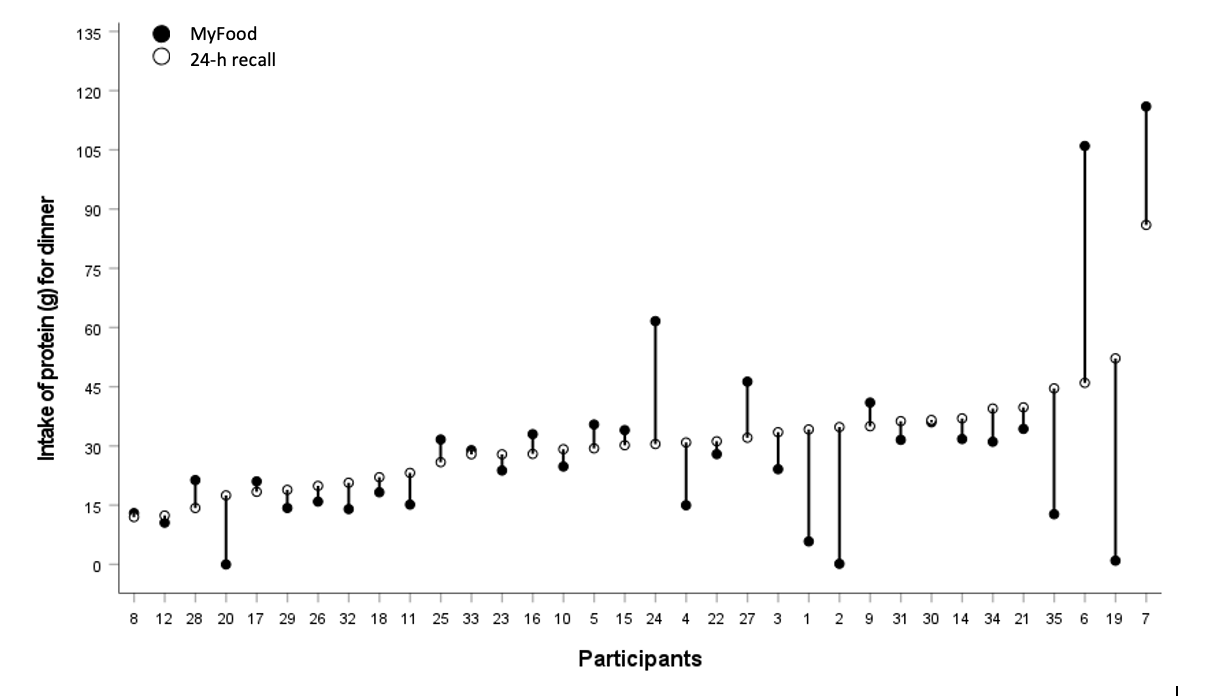


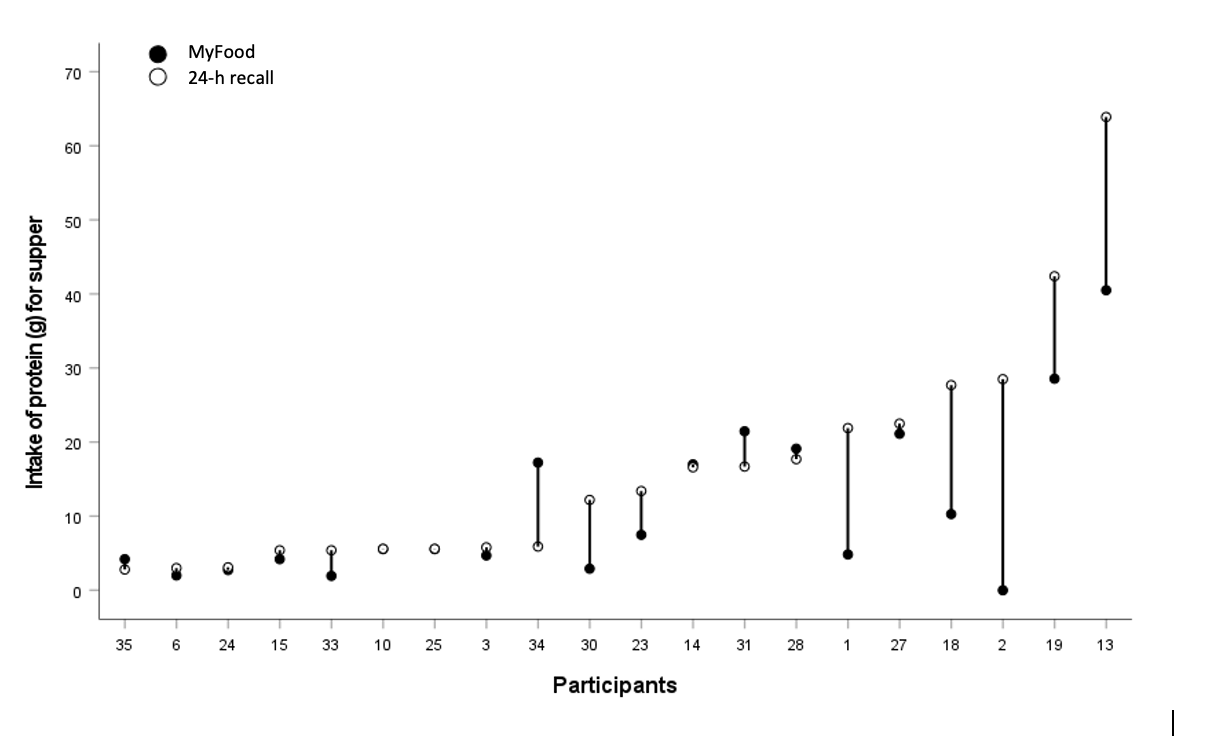


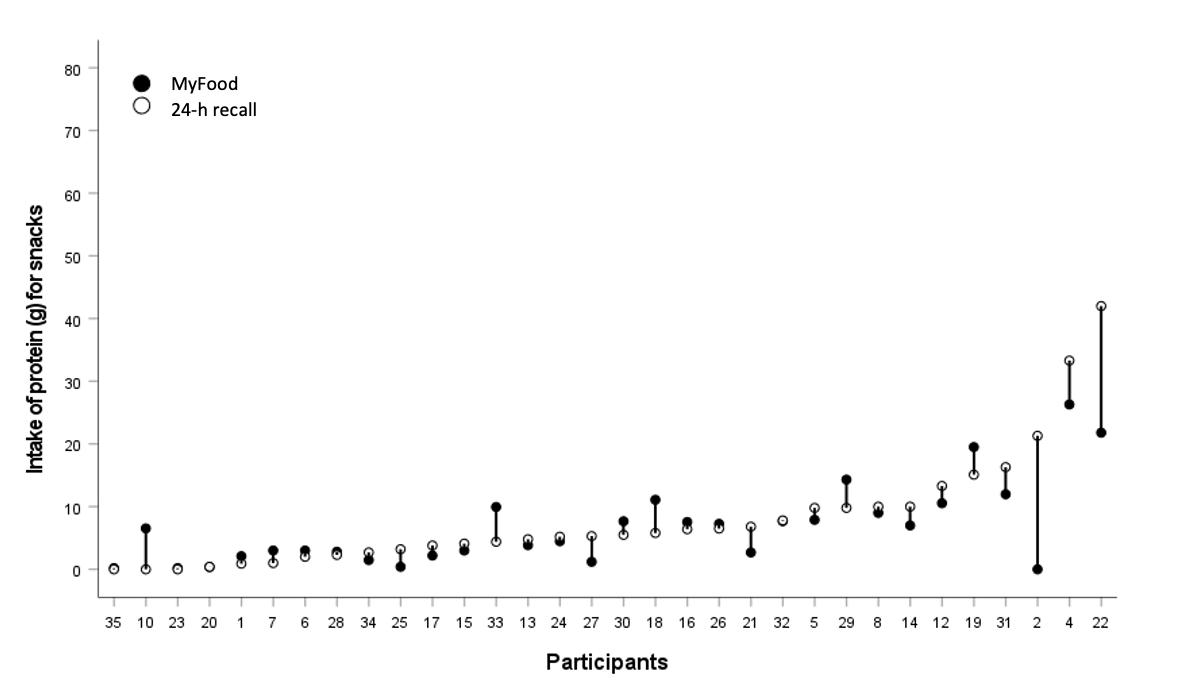


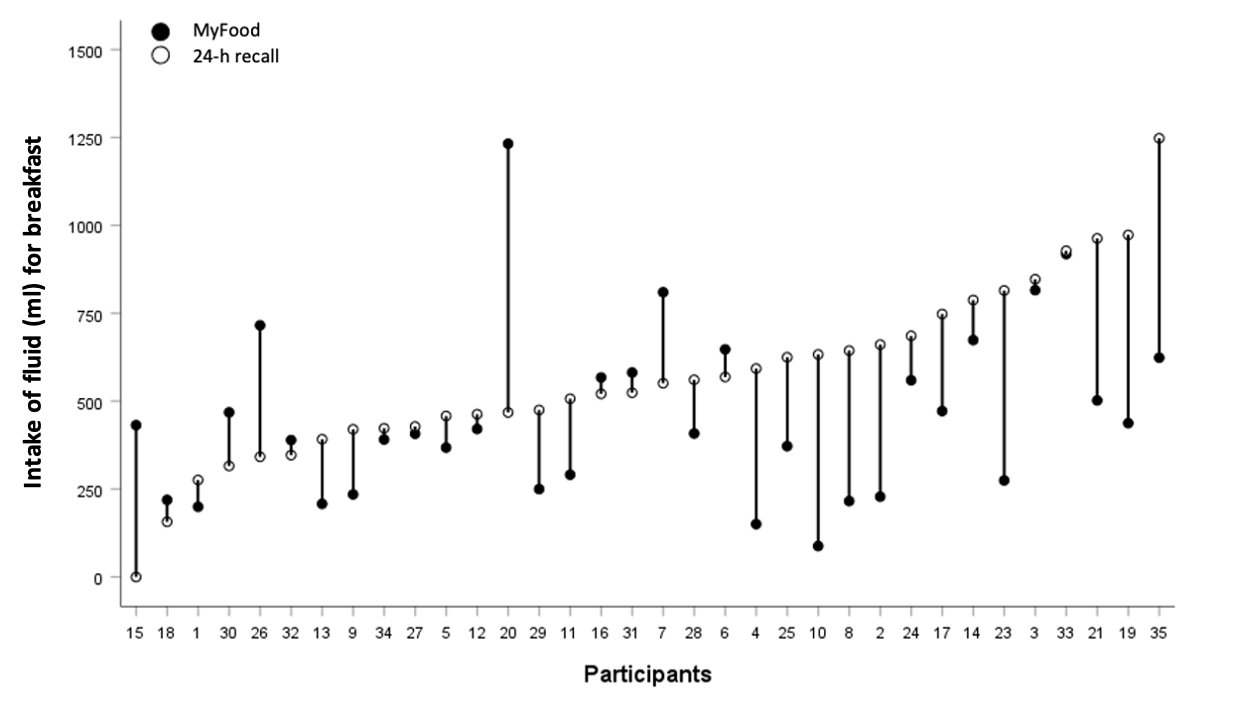


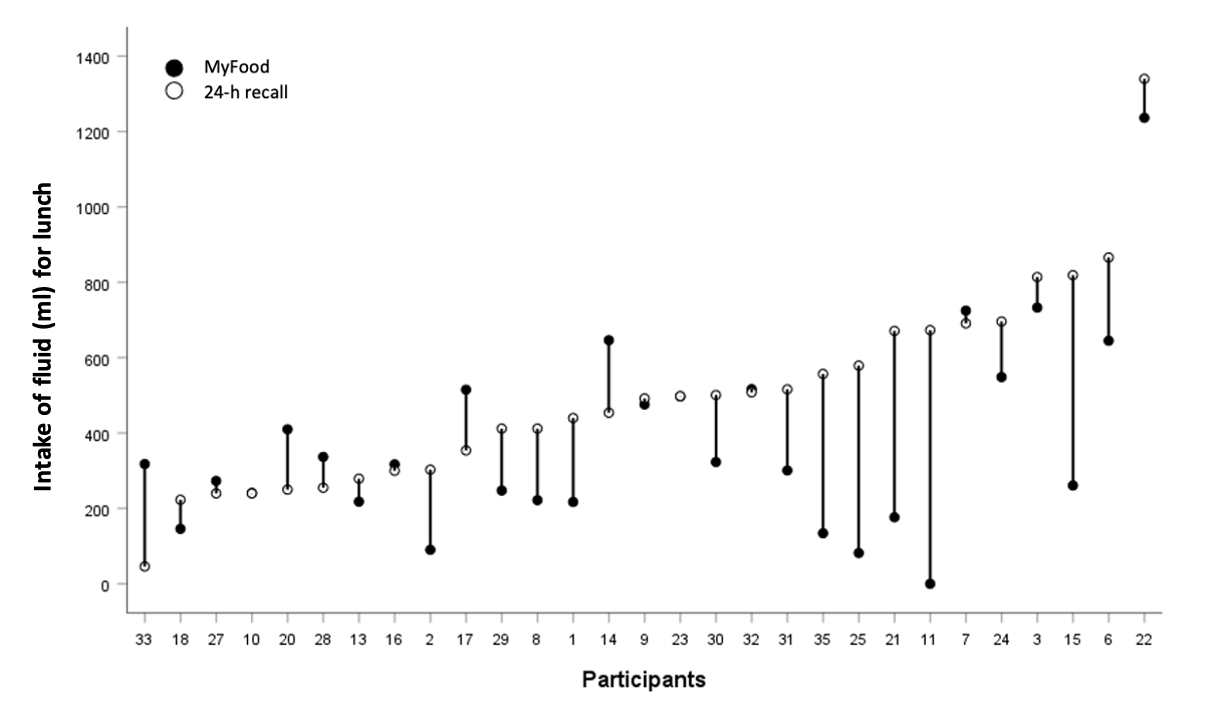


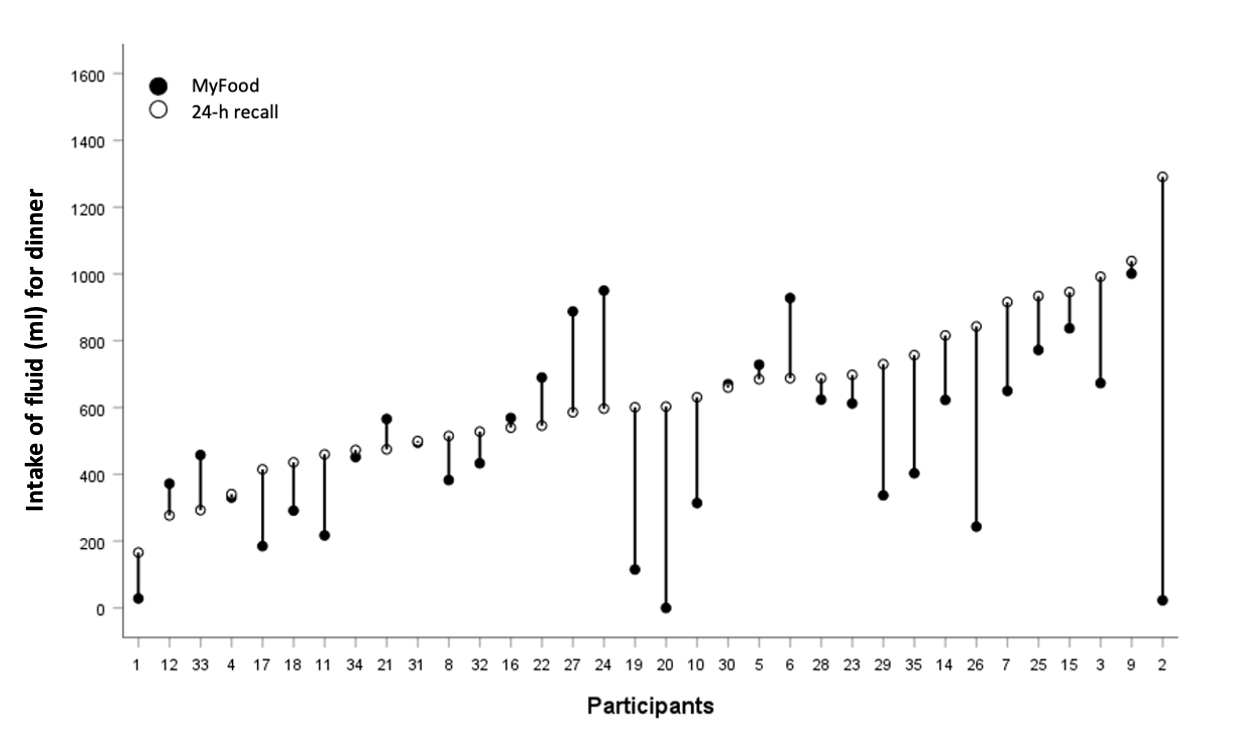


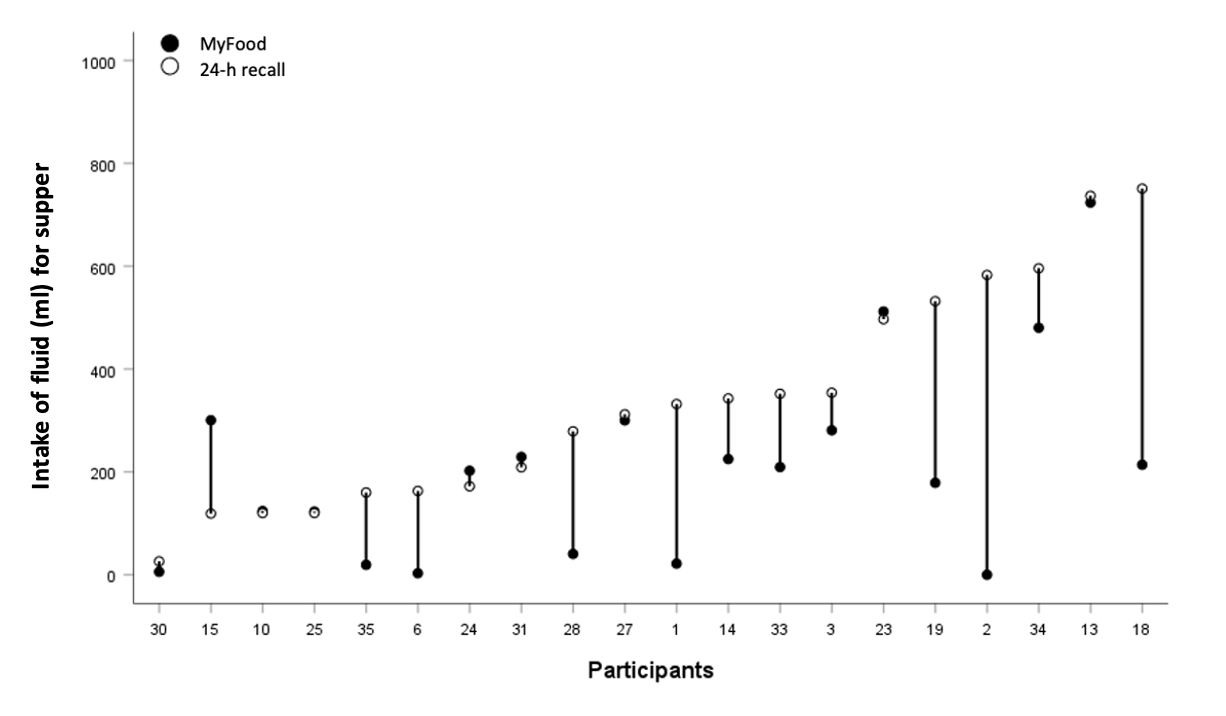


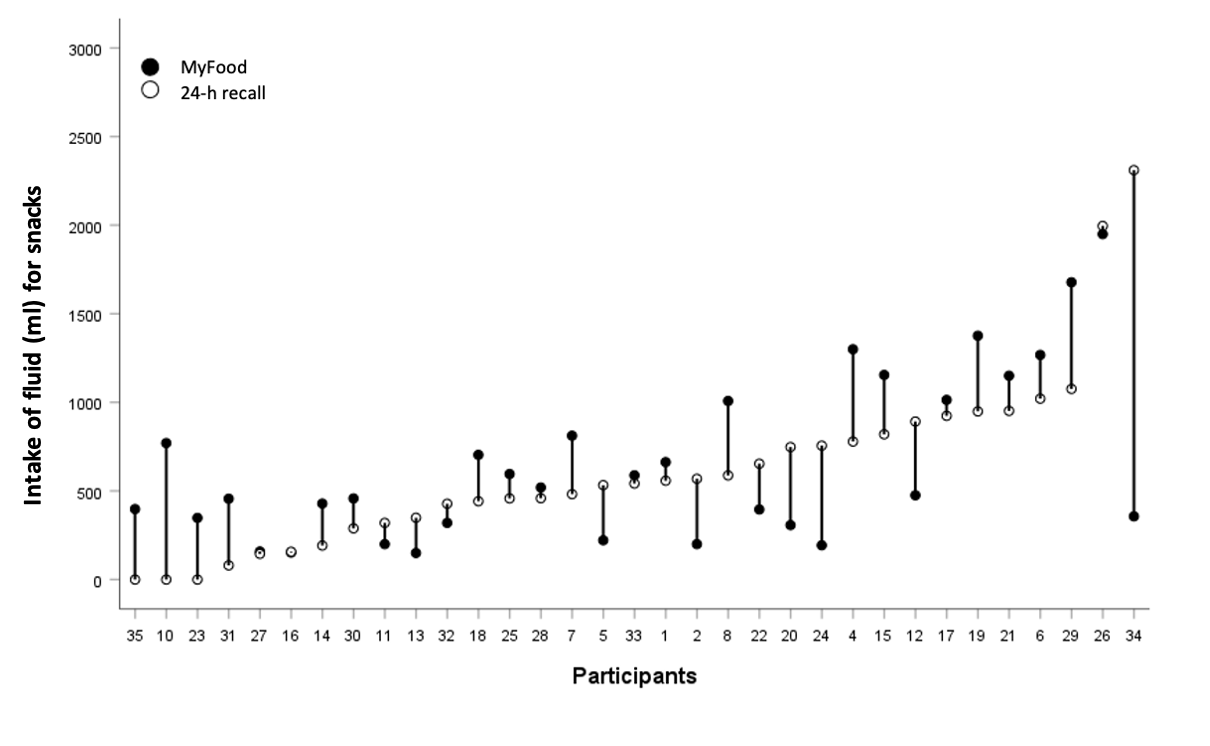

Supplement: Multimedia Appendix 1 [file mhealth_v11i1e45079_app1.docx]

Drop-plots for intake of energy, protein, and fluids for each meal on comparison day 2


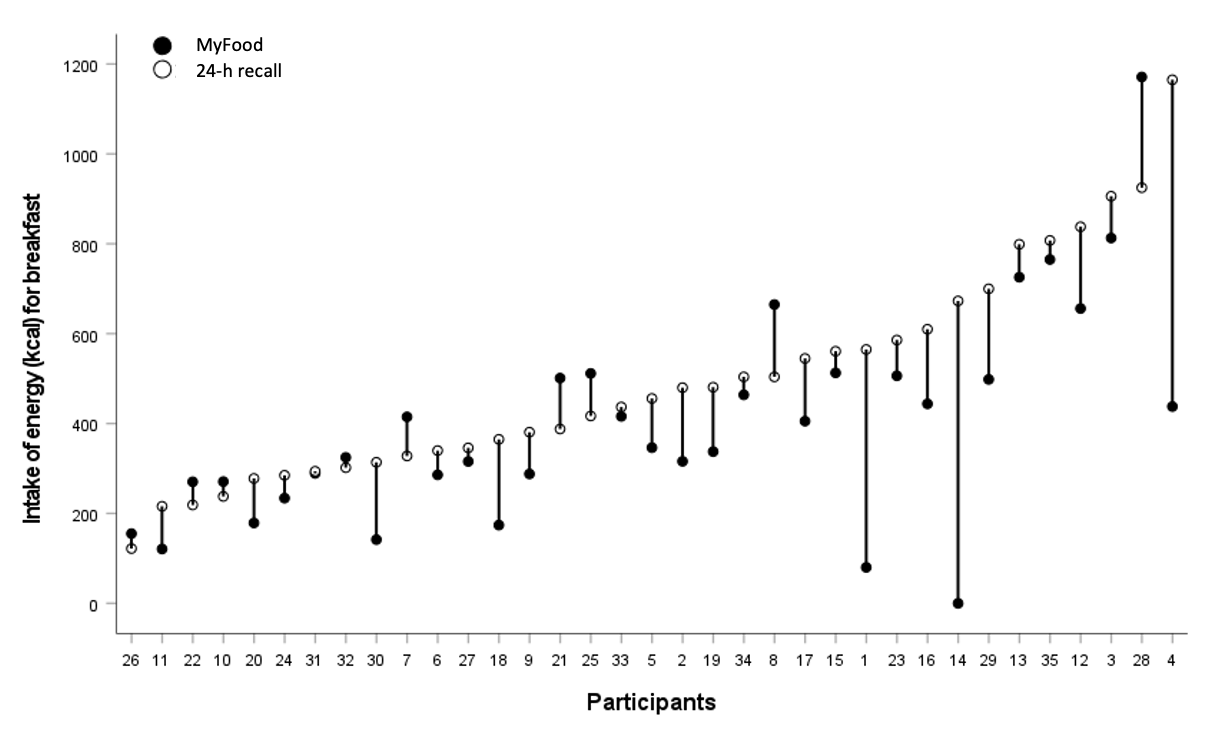


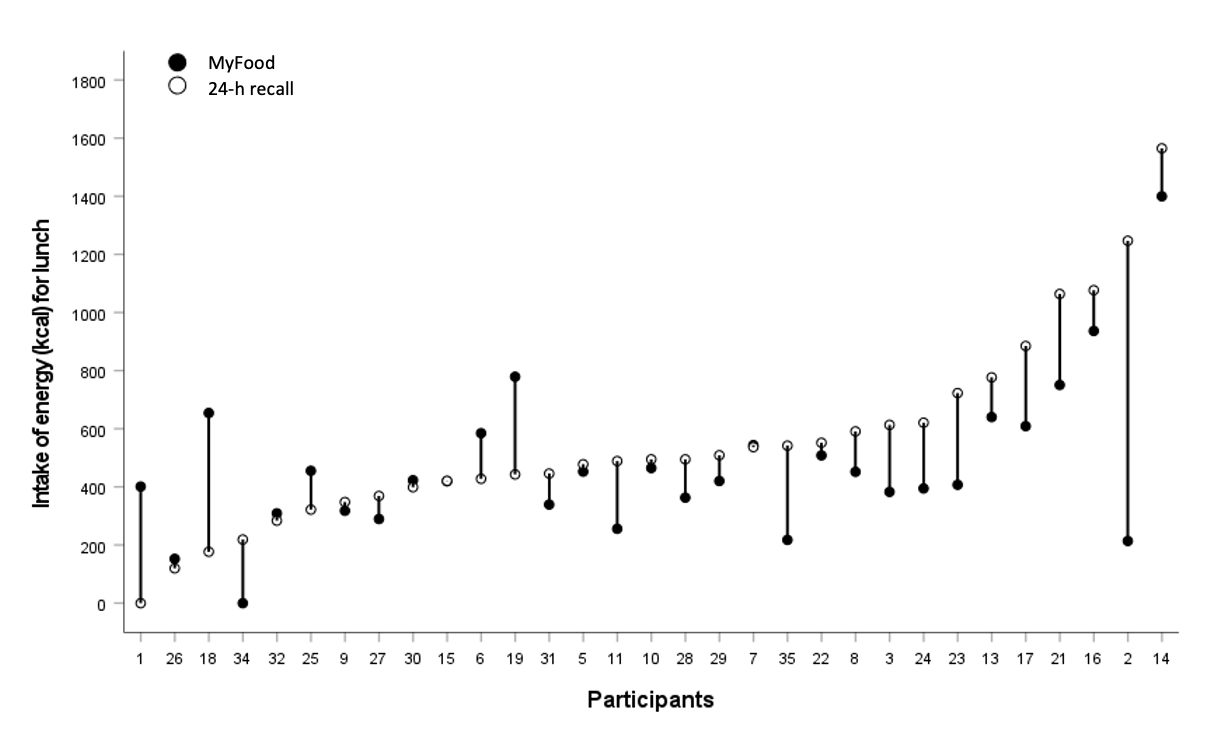


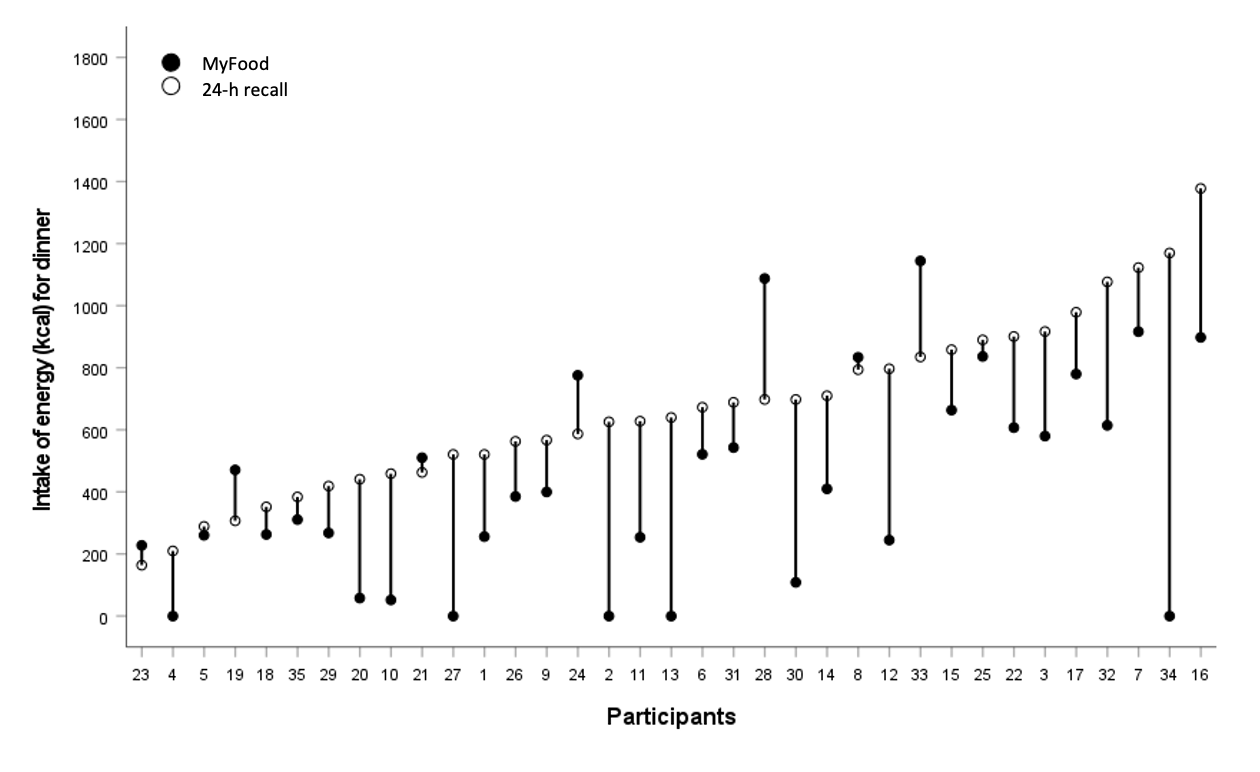


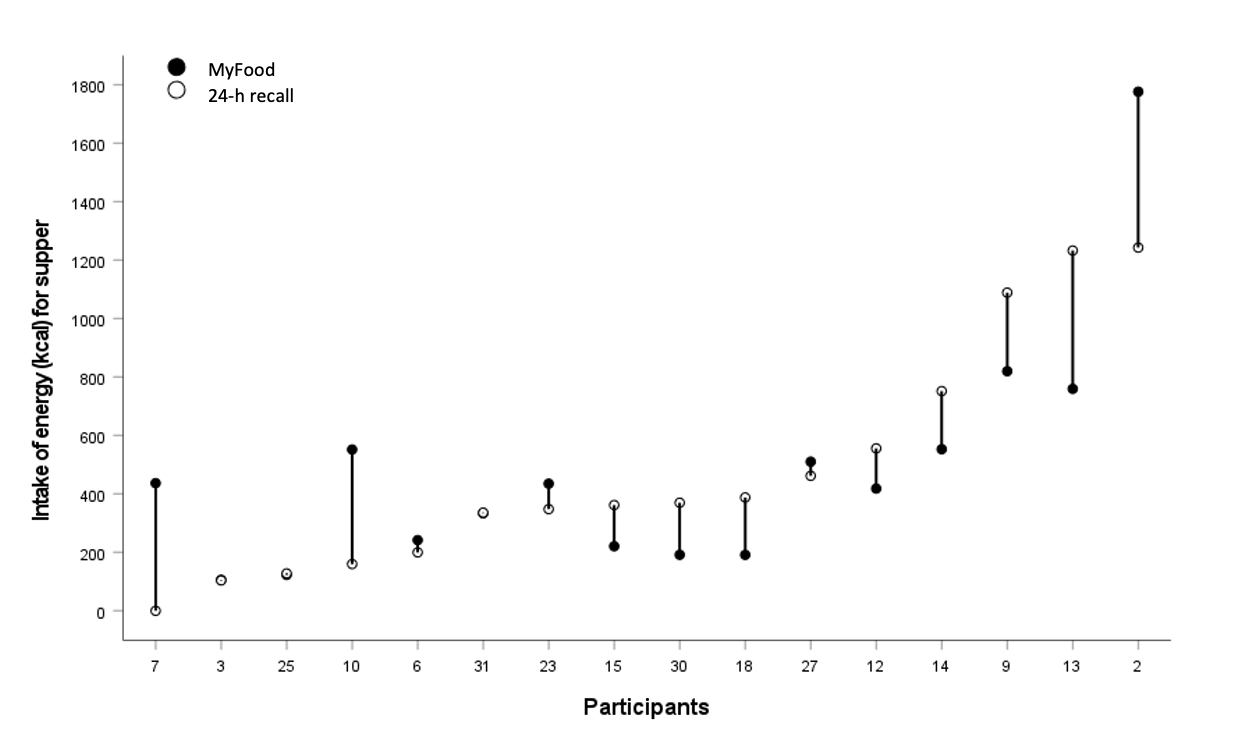


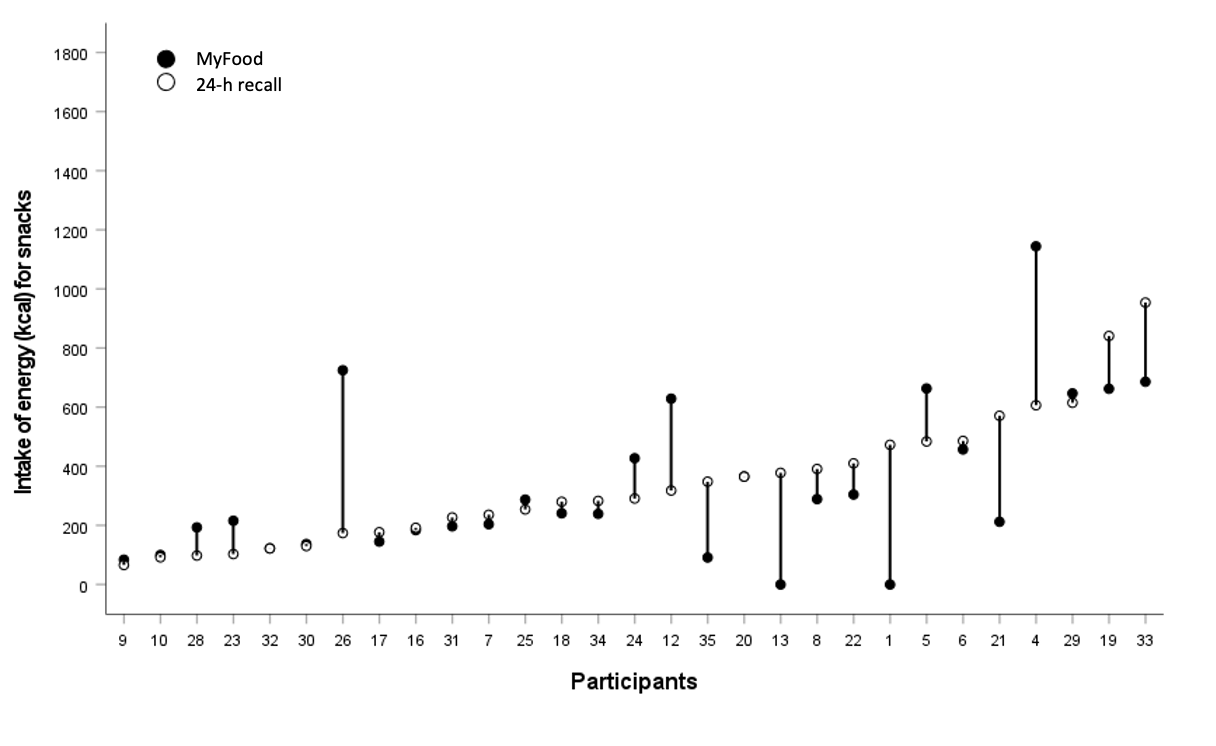


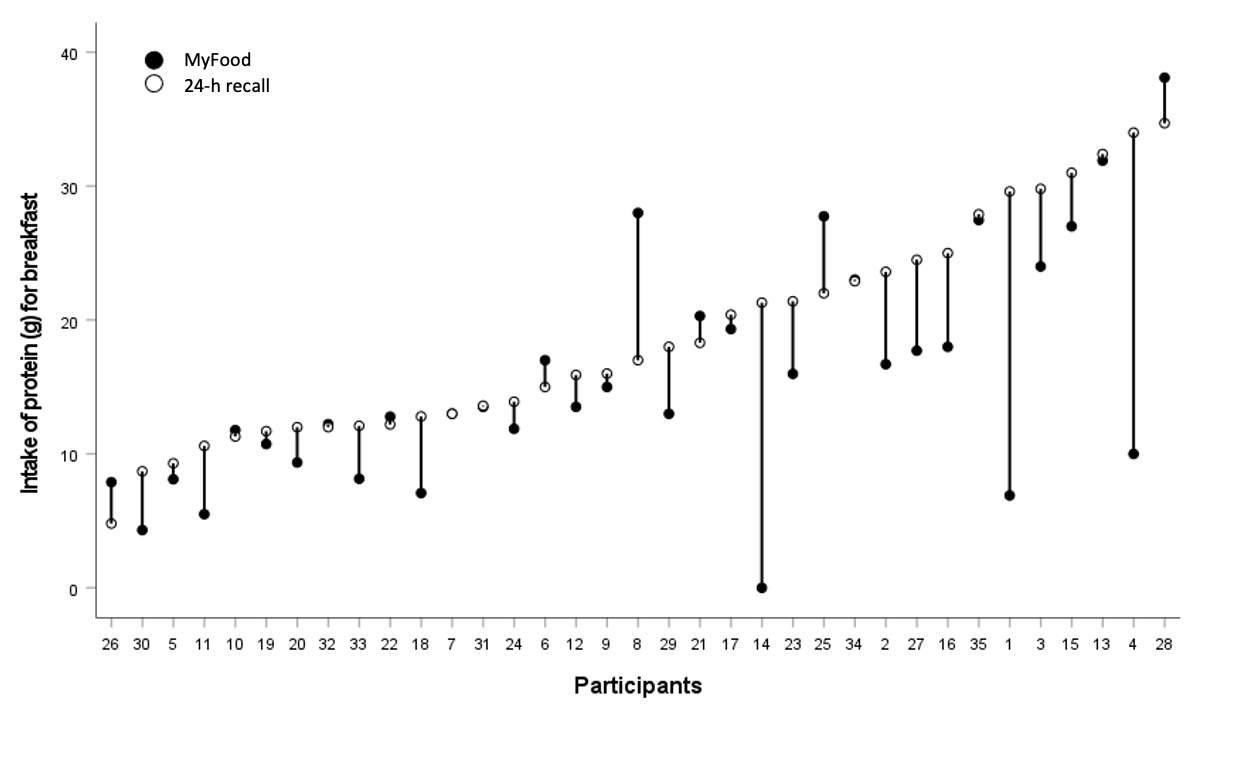


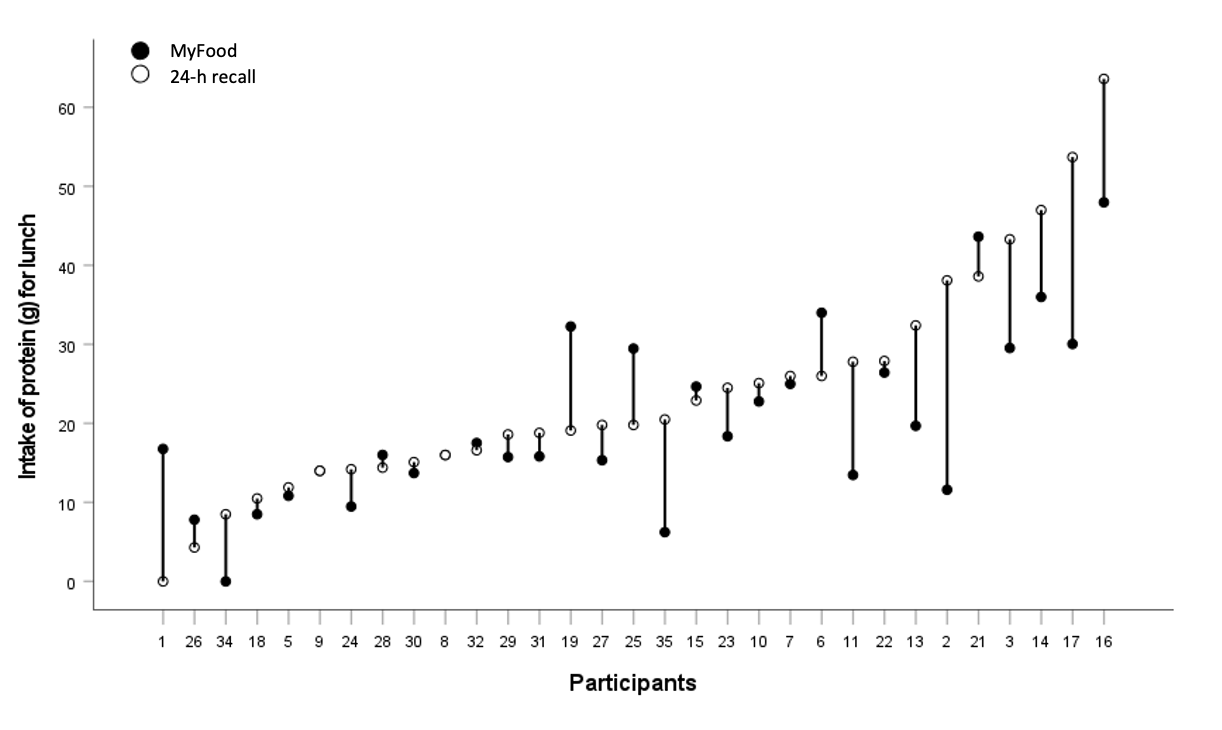


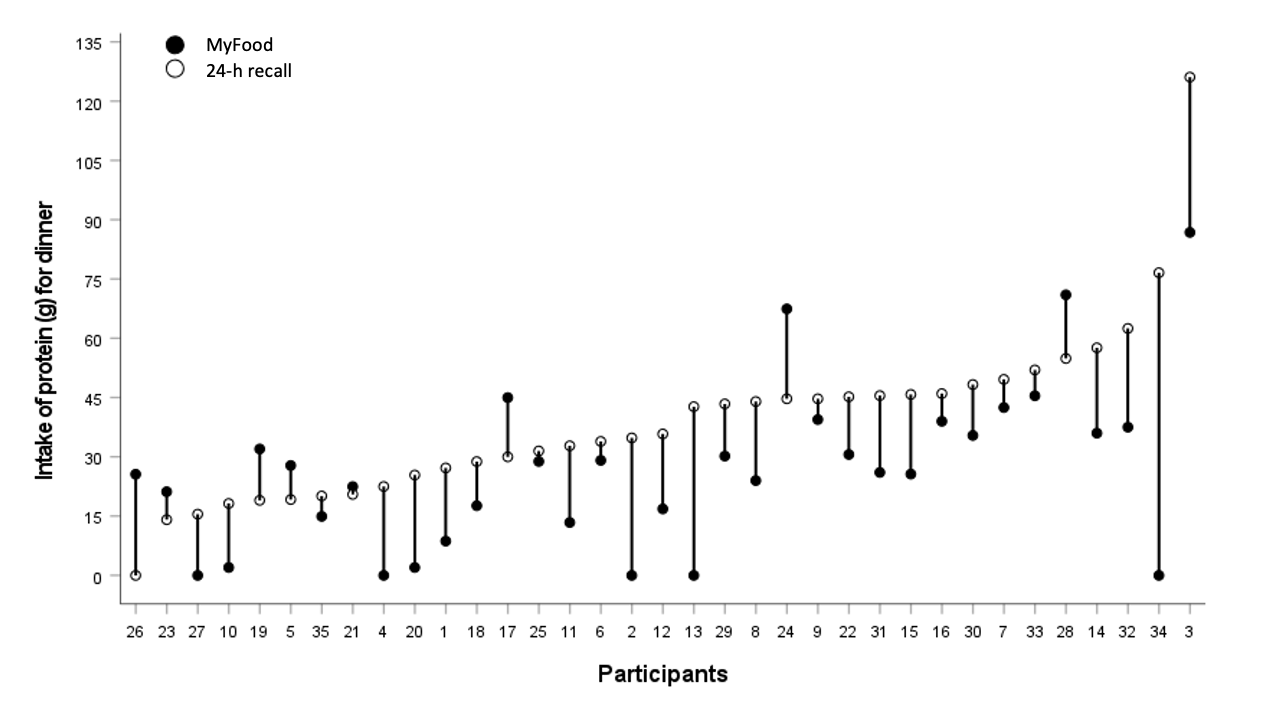


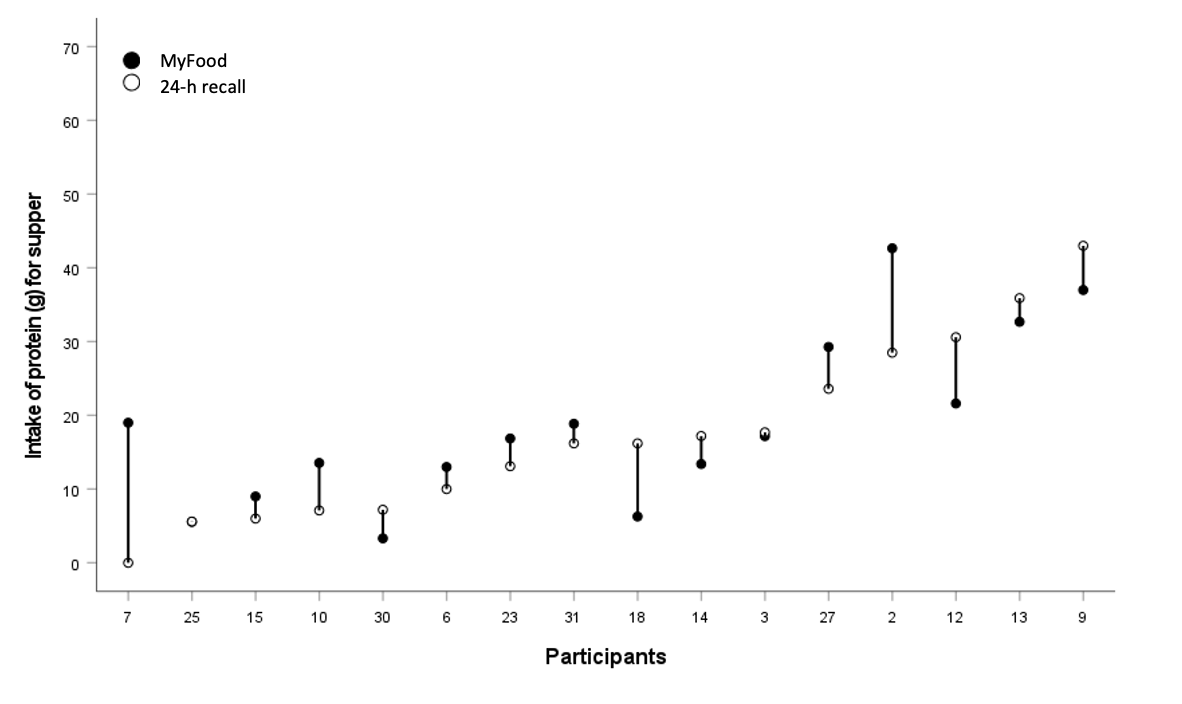


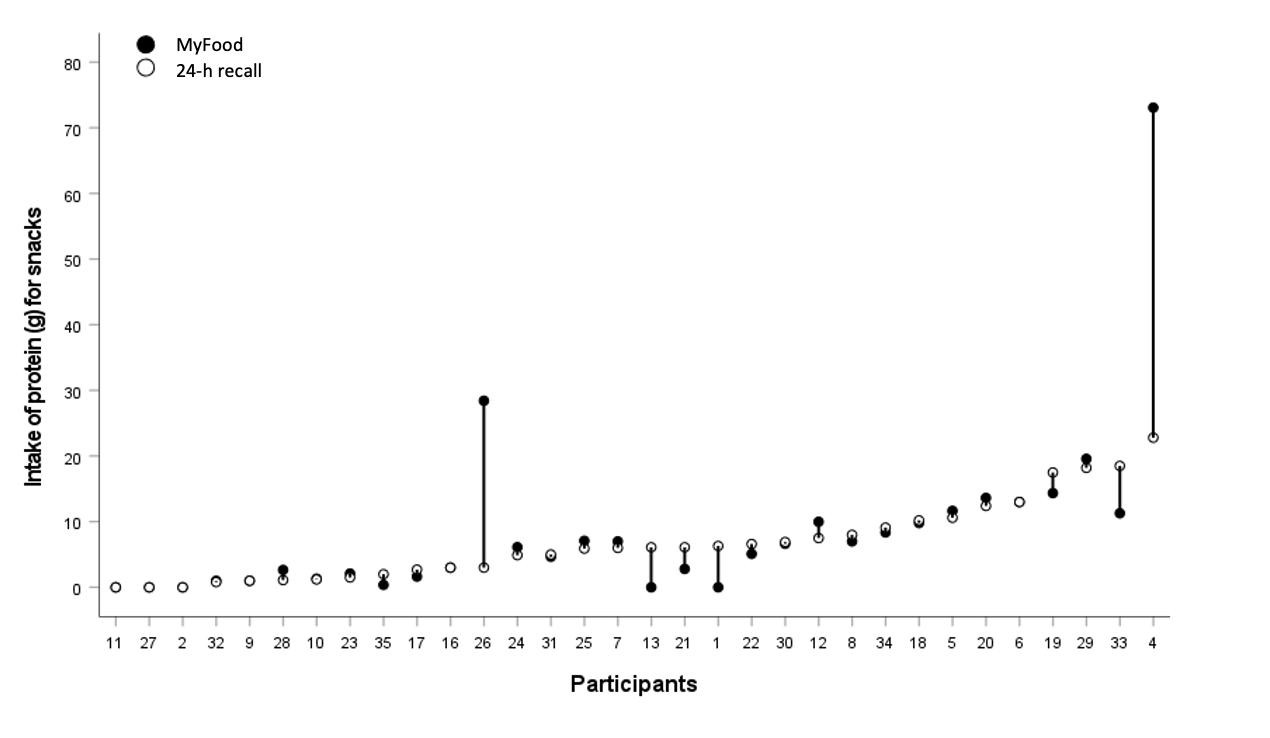


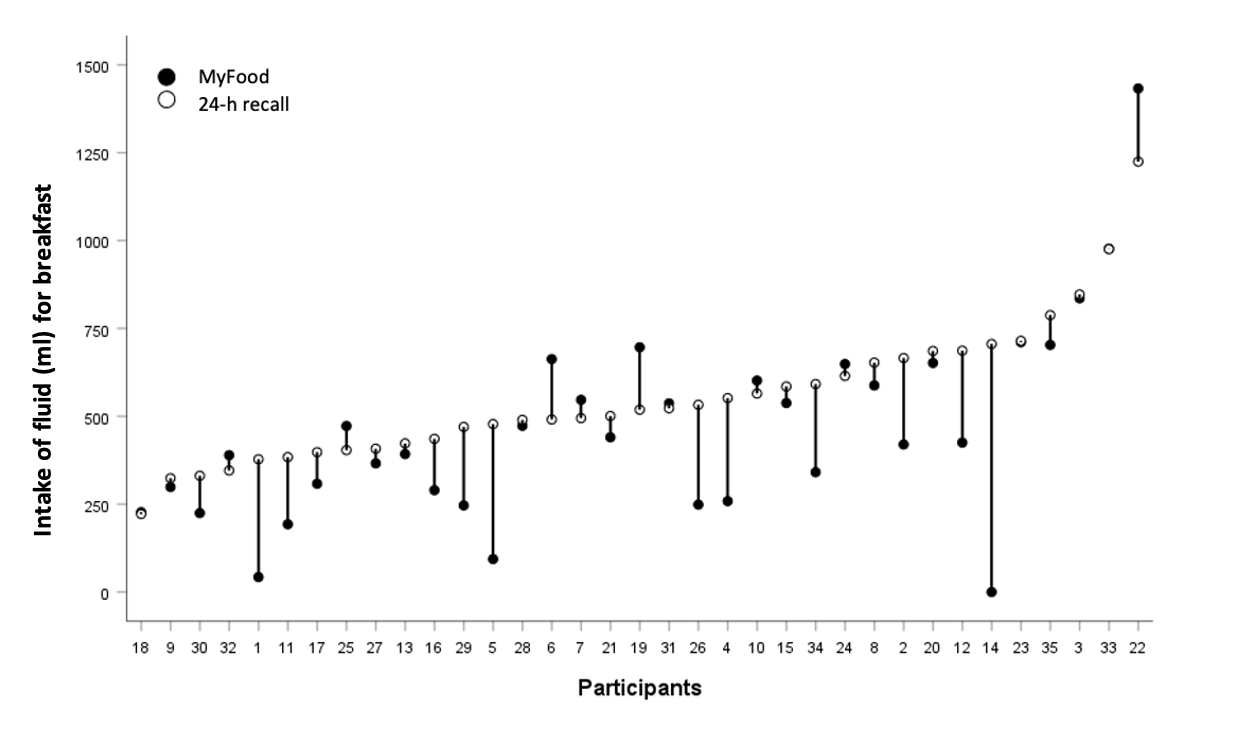


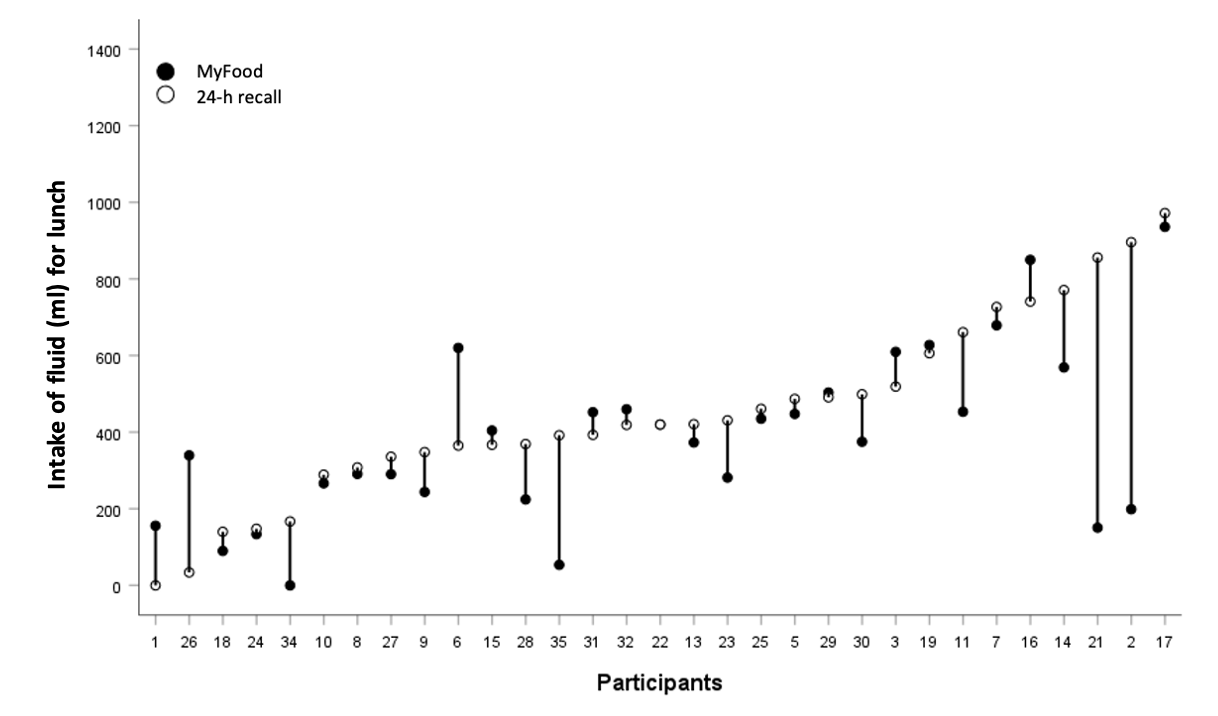


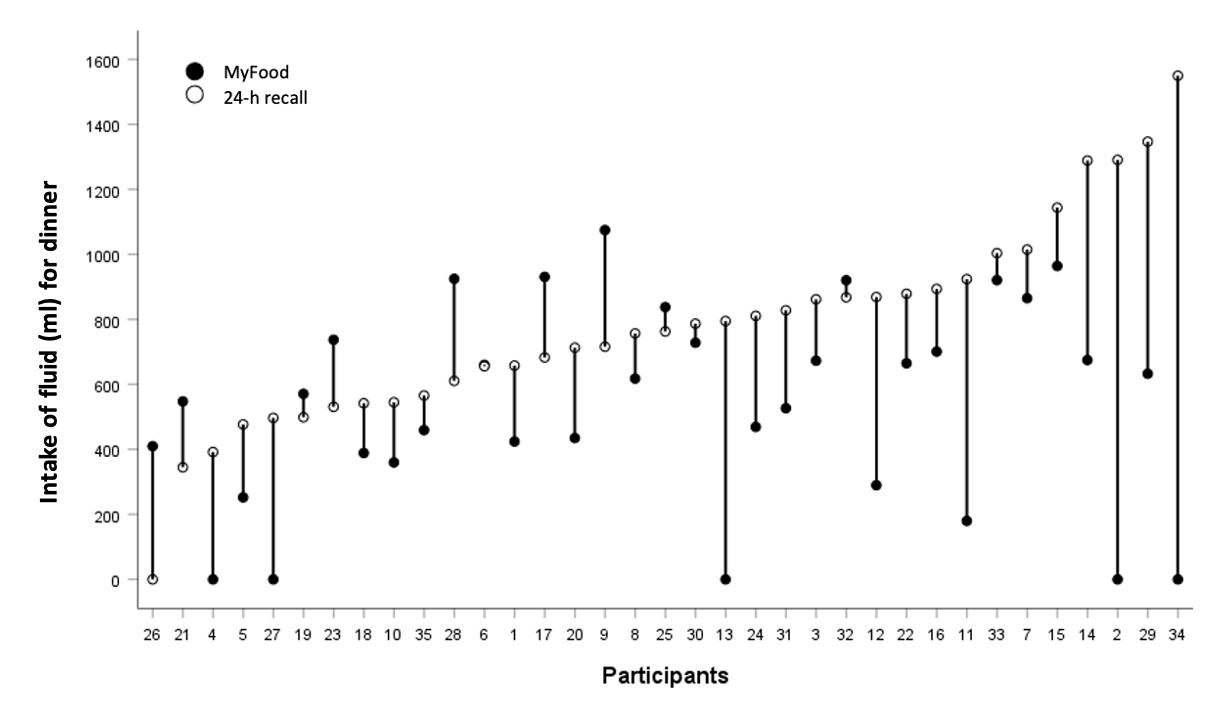


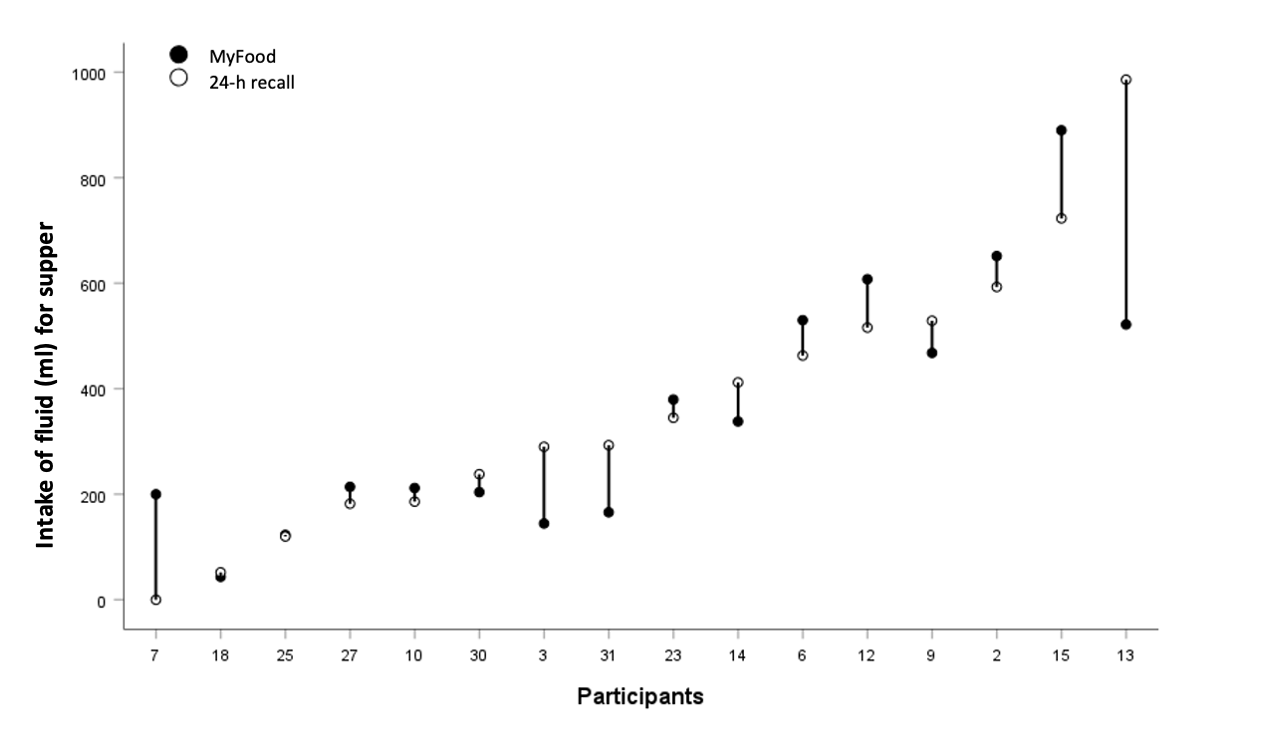


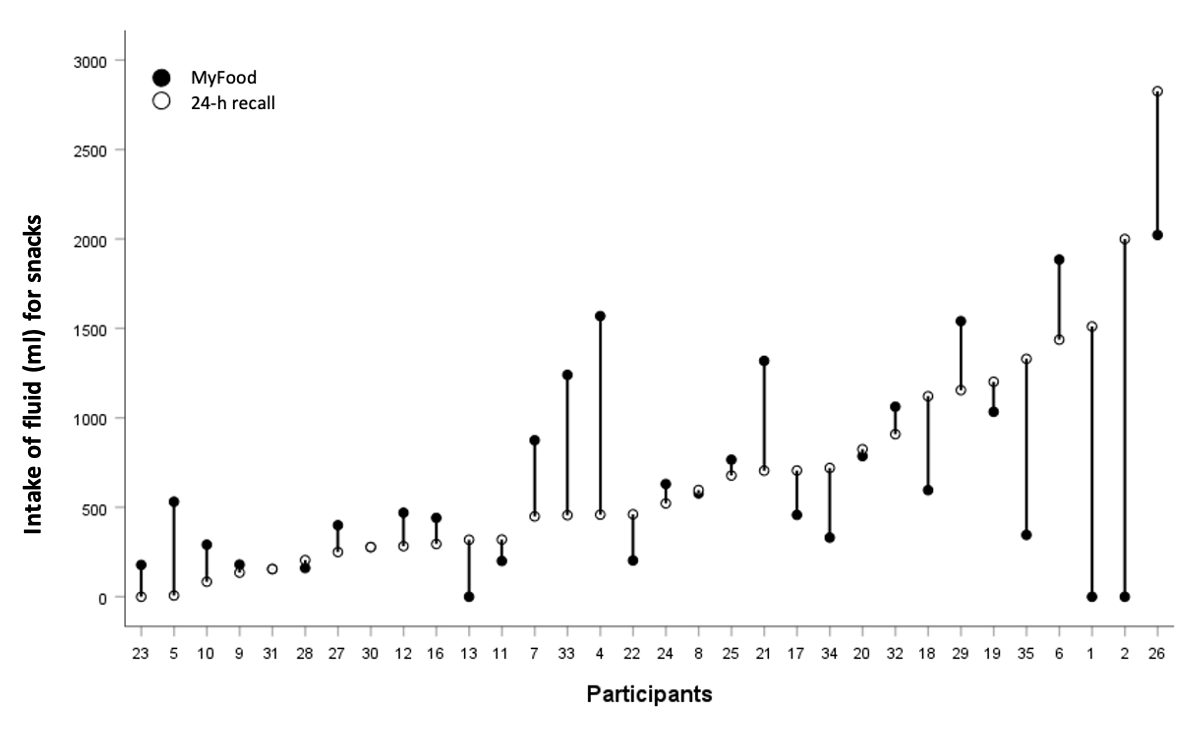

Supplement: Multimedia Appendix 2 [file mhealth_v11i1e45079_app2.docx]

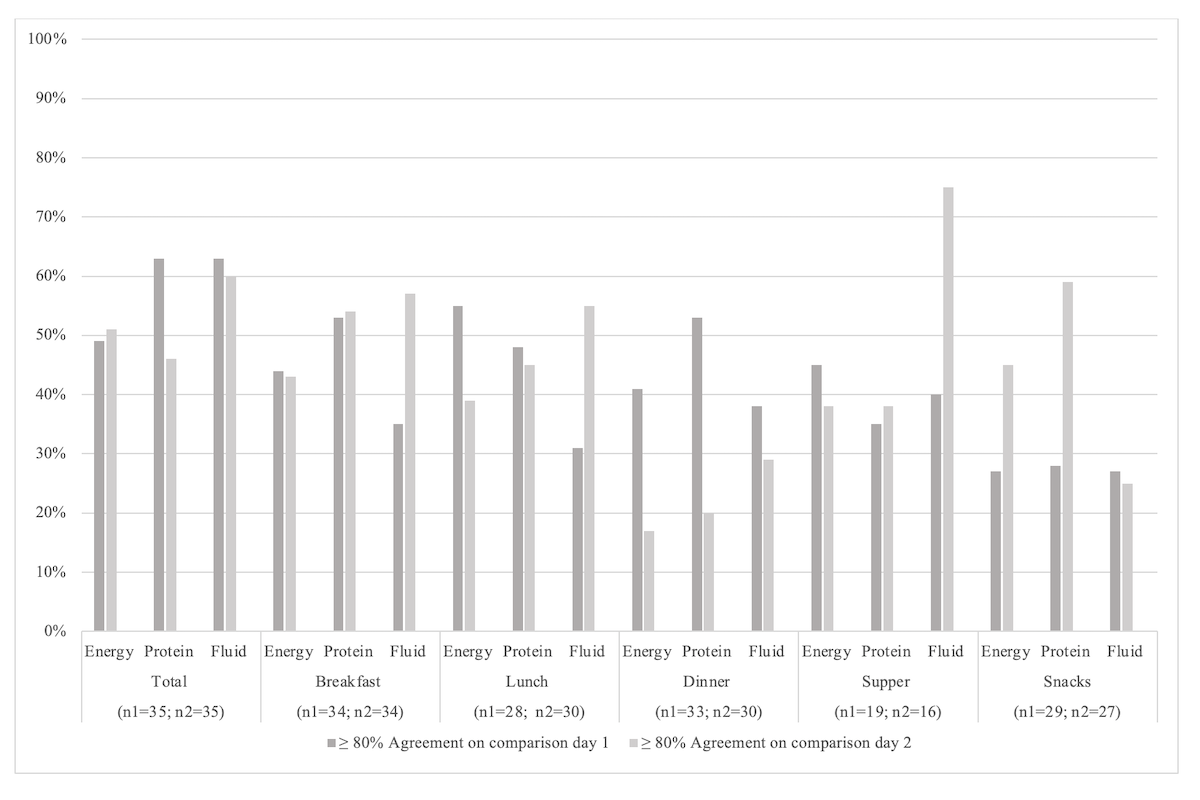

Supplement: Multimedia Appendix 3 [file mhealth_v11i1e45079_app3.png]

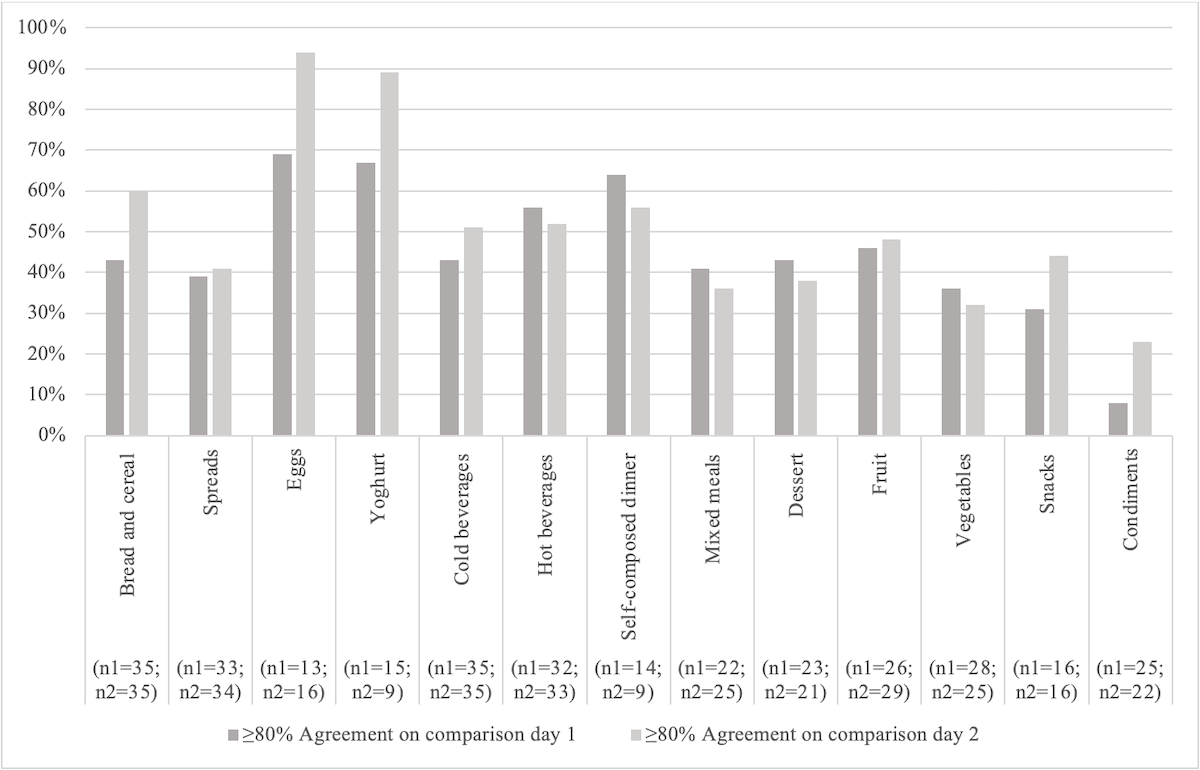

Supplement: Multimedia Appendix 4 [file mhealth_v11i1e45079_app4.png]

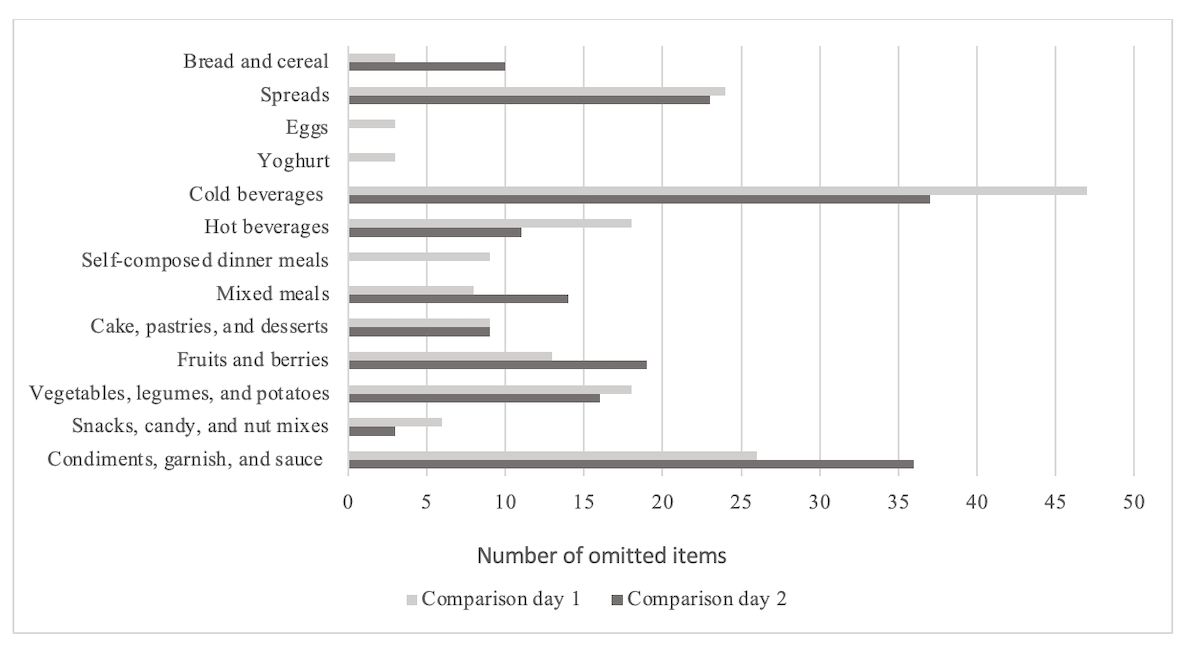

Supplement: Multimedia Appendix 5 [file mhealth_v11i1e45079_app5.png]
